# Detection and quantification of rumen methanogens using F_420_ autofluorescence profiling with spectral flow cytometry

**DOI:** 10.1128/aem.01416-25

**Published:** 2026-02-09

**Authors:** Sofia Khanum, Joanna M. Roberts, Maria M. Della Rosa, Rechelle Sage, Peter M. Reid, Priya Soni, Stefanie Bagley, Stefan Muetzel, Peter H. Janssen, D. Neil Wedlock

**Affiliations:** 1 Lucidome Bio Ltd, Wellington, New Zealand; 2 AgResearch Group, Bioeconomy Science Institute, Palmerston North, New Zealand; 3 Flowjoanna Tāpui Ltd, Palmerston North, New Zealand; University of Nebraska-Lincoln, Lincoln, Nebraska, USA

**Keywords:** methanogens, rumen, spectral flow cytometry, F_420 _autofluorescence

## Abstract

Methanogenic archaea that reside in the rumen of sheep, cattle, and other ruminants generate 16% of global emissions of methane, a potent greenhouse gas. The majority of rumen methanogens belong to species that display readily observable autofluorescence due to their intracellular co-factor, F_420_. We developed a spectral flow cytometry method to directly quantify autofluorescent methanogens in the complex environment of the rumen. Rumen samples contain feed particles with natural autofluorescence signatures that overlapped those of F_420_-containing methanogens. Spectral unmixing using natural autofluorescence signatures allowed us to distinguish methanogens from other autofluorescent particles and to quantify both cultured methanogens in buffer and native methanogens in rumen content samples over a concentration range from 4 × 10^4^ to 4 × 10^7^ cells/mL. The methanogen signal was absent in microbial cultures known to lack F_420_ and in rumen content samples treated with sodium borohydride (NaBH_4_), which reduces F_420_ fluorescence. We showed a strong relationship between the number of autofluorescent methanogens in rumen content samples and methane yields in cattle and sheep treated with a methanogen inhibitor. We also assessed the impact of sample fixation on the spectral profiles of methanogen cells and showed that rumen samples stored at 4°C for up to 3 days remain suitable for enumeration. Our data thus demonstrate a new spectral flow cytometry method that can be used for rapid quantification of autofluorescent methanogens in rumen content samples.

**IMPORTANCE:**

Production of methane, a potent greenhouse gas, by methanogenic archaea in cattle, sheep, and other ruminants contributes around 16% of global methane emissions. Methane mitigation strategies are essential to respond effectively to the challenge of climate change, and many mitigations target rumen methanogens directly. In this study, we developed a method based on methanogen autofluorescence to identify and quantify methanogens within the complex rumen environment containing plant material that also fluoresces. The overlapping autofluorescence signals from methanogens and plant material can be resolved by applying spectral unmixing in spectral flow cytometry. This technique provides a rapid, practical approach for detecting and quantifying methanogens directly in rumen samples without the need for staining or additional fluorescent dyes or reagents. It provides a valuable tool to assess the impact of mitigation technologies. The method should also allow direct measurement of antibody binding to methanogens or determination of co-location of methanogens with other microbes.

## INTRODUCTION

Methanogenic archaea (methanogens) that reside in the rumen of cattle, sheep, and other ruminants use hydrogen, formate, and methyl compounds derived from rumen fermentation to form methane (1, 2) via a process known as methanogenesis. This methane is expelled by the animal into the atmosphere. Methane emissions from ruminants contribute 16% of global greenhouse gas emissions worldwide and also represent a potential loss of feed energy to the animal (3–5). Various methane mitigation strategies are being developed, including selection of low methane-producing animals, use of specific forages that result in lower methane emissions than those standardly fed, use of dietary feed additives that modify the ruminal fermentation, and use of probiotics (6–8). In addition, there has been progress in the development of vaccines and chemical inhibitors that target the methanogens directly to reduce methane emissions from livestock (9, 10).

Ruminal methanogens fall broadly into two groups (11): members of the order *Methanobacteriales* that are characterized by their fluorescence due to cofactor F_420_ (8-hydroxy-5-deazaflavin; [12]), and members of the order *Methanomassiliicoccales*, that do not display F_420_ fluorescence (13). In a global survey of rumen methanogens, members of *Methanobacteriales* were found to make up 85.1% of rumen methanogens (14). Because this group is phylogenetically coherent, they are a good target for a vaccine to reduce methane emissions from farmed ruminants (15).

A range of different techniques is available to quantify the abundance of methanogens in environmental samples. These are primarily based on methods that target the broadly phylogenetically informative 16S *rRNA* gene of methanogens (16) or the functional *mcrA* gene coding for the α-subunit of the methyl coenzyme M reductase (17). Broader metagenomics analyses to assess the microbial composition of samples can also give insights into methanogen populations (18–20). Fluorescence microscopy based on the fluorescence of F_420_ has been used as a direct approach to quantify methanogens in complex microbial communities (21). However, the microscopy technique is labor- and time-intensive, even with continued developments in apparatus automation and image analysis (22). To minimize the uncertainty of the count caused by statistical counting error, a large number of events must be included, which can be challenging with microscopy (23). In addition, this method requires the use of strictly standardized counting procedures and thorough cross-calibration among individual users to ensure reproducible results.

Single-cell labeling by fluorescent *in situ* hybridization (FISH) has been used to quantify methanogens (24) and can be combined with flow cytometry. Flow cytometry has also identified and quantified methanogens based on their intrinsic fluorescence due to F_420_ (12, 25, 26). F_420_ displays a distinct blue fluorescence with an excitation maximum at 420 nm in its oxidized state and can be applied to quantify the majority of rumen methanogens (25). The method does, however, need to discriminate between the fluorescence due to F_420_ and overlapping fluorescent spectra found in plant constituents (27) present in rumen samples.

A flow cytometry approach to cell counting should enable direct quantification of cells of autofluorescent methanogens in rumen samples using their F_420_ fluorescence and could be coupled with fluorophore-labeled antibodies, dyes to stain nucleic acids, or rRNA-directed FISH probes to study antibody binding or co-location with other microbes. Flow cytometry has advantages for cell quantification, including usability over a wide dynamic range of microbial cell concentrations, that is, 10^3^–10^8^ cells/mL, high fluorescence sensitivity capable of statistically significant cell counts measuring thousands of cells per second, relatively fast turnaround time, and the potential for simple methodologies (28–30). Flow cytometers use a variety of light beams to examine each passing cell and measure optical diffraction (light scatter) as well as a range of fluorescent wavelengths using discrete bandpass (BP) emission filters.

Spectral flow cytometry handles fluorescent signals by stitching together a full spectrum profile from BP filters that span the major part of the emission spectrum. Spectral flow cytometry uses spectral unmixing instead of traditional compensation to separate overlapping fluorescence signals. It captures the full emission spectrum of each fluorophore and applies algorithms to distinguish their contributions. By measuring the full spectrum of each cell, the natural autofluorescence profile can be detected and can be sufficient to capture biological features without the need for staining (31).

In this study, we developed a spectral flow cytometry protocol based on the intrinsic fluorescence of the methanogen cofactor F_420_ to detect and quantify native F_420_-containing methanogen populations within the complex environment of rumen content samples from cattle and sheep.

## MATERIALS AND METHODS

### Methanogen isolates and culture conditions

Methanogen isolates used in this study were *Methanobrevibacter ruminantium* M1 (32), *Methanobrevibacter boviskoreani* AbM4 (33), *Methanobrevibacter millerae* SM9 (34), *Methanobrevibacter olleyae* 229/11 (35), *Methanobrevibacter* sp. D5 (36), *Methanosphaera* sp. ISO3-F5 (36), and *Methanomassiliicoccaceae* isolate ISO4-G1 (36). Isolates were revived from frozen stocks and grown anaerobically in BY^+^ medium in 10 mL volumes in Hungate tubes (Bellco Glass, Vineland, New Jersey, USA) or 60 mL volumes in 125 mL serum vials (Bellco Glass) under a CO_2_ headspace and sealed using butyl rubber stoppers to which H_2_ gas was added to 0.8 bar pressure over pressure. Cultures were incubated at 39°C in the dark, either upright statically (Hungate tubes) or on their sides on a shaking platform at 50 rpm (serum vials). BY^+^ medium was prepared by mixing solution A (17% [vol/vol]), solution B (17% [vol/vol]), rumen fluid (30% [vol/vol]), NaHCO_3_ (5 g/L), yeast extract (2 g/L), 5 drops of resazurin (0.1% [wt/vol]), L-cysteine-HCl (0.5 g/L), and selenite/tungstate solution (37) (0.1% [vol/vol]). All components except L-cysteine-HCl were mixed thoroughly, boiled under O_2_-free 100% CO_2_, and cooled in an ice bath while being bubbled with 100% CO_2_. Once the solution had cooled, the L-cysteine-HCl was added, and the media were dispensed into culture vessels under 100% CO_2_. These were sterilized by autoclaving for 20 min at 121°C and stored in the dark for at least 24 h before use. Salt solution A was prepared by mixing NaCl (6 g/L), KH_2_PO_4_ (3 g/L), (NH_4_)_2_SO_4_ (1.5 g/L), CaCl_2_·2H_2_O (0.79 g/L), and MgSO_4_·7H_2_O (1.2 g/L) in distilled water. Salt solution B was prepared by dissolving K_2_HPO_4_·3H_2_O (7.86 g/L) in distilled water.

Cells in late-logarithmic to early stationary-phase cultures were harvested by centrifugation at 5,000 *g* for 5 min. Cell pellets were washed three times with phosphate-buffered saline (PBS, 10 mM, pH 7.3) for *in vitro* methanogen quantification experiments using flow cytometry, qPCR, and microscopy. A mixed methanogen sample (Mix5) was prepared by mixing an equal number of cells of isolates M1, AbM4, SM9, D5, and 229/11.

Rumen bacterial isolates used in this study were *Butyrivibrio proteoclasticus* B316 (38), *Ruminococcus albus* 7 (39), *Selenomonas ruminantium* HD4 (40), *Aristaeella hokkaidonensis* R-7 (41), *Olsenella umbonata* Hun279 (42), and *Prevotella* sp. C21a (43). All bacterial isolates were grown in Hungate tubes in BY^+^ medium as described above without added selenite/tungstate solution or H_2_ but containing 20 mM glucose as substrate, except for *S. ruminantium* HD4 and *R. albus* 7, which used 30 mM cellobiose as their energy source. Cells were grown statically at 39°C to late-logarithmic to early stationary phase and harvested, washed, and resuspended as described above.

### Methanogen inhibition in cattle and sheep

Twenty-three 12- to 15-month-old beef cross heifers with an average live weight of 330 kg were fed fresh ryegrass (*n* = 11) or ryegrass-based baleage (*n* = 12). Of these animals, 12 were administered a methanogen inhibitor in their feed at a level high enough to fully inhibit their methane emissions, 3 animals received an inhibitor dose for partial methane inhibition (20%–50% lower methane yield), 6 received an ineffective dose of inhibitor to decrease methane emissions, and 2 animals were not administered the inhibitor (Table S1).

Ten 6-month-old lambs with an average live weight of 40 kg were fed fresh ryegrass. Seven of these lambs received a dose of the same inhibitor in their feed sufficient to fully inhibit their methane emissions, while the remaining three lambs did not receive the inhibitor (Table S2). The identity of the inhibitor used in both the cattle and sheep studies is commercially sensitive and so is not disclosed.

### Measurement of methane emissions and collection and treatment of rumen content samples

Methane emissions from the cattle and sheep were quantified in open-circuit respiration chambers at the New Zealand Ruminant Methane Measurement Centre (AgResearch, Palmerston North, New Zealand) over 2 days for each animal (44). Rumen content samples were collected immediately after the animals left the respiration chambers, before morning feeding to ensure a more homogeneous microbial suspension, via oral stomach tubing using a 150 mL syringe coupled to a flexible tube. The concentrations of rumen volatile fatty acids and alcohols (ethanol and propanol) were determined by gas chromatography (45). A 2 mL aliquot of rumen content sample was filtered through a 100 µm cell strainer (Corning, Hamburg, Germany) and diluted 1:50 in PBS prior to performing spectral flow cytometry analysis. A 200 µL aliquot of each filtered rumen content sample was stored at −20°C for subsequent DNA extraction and qPCR.

### Preparation of feed and rumen fluid samples for spectral flow calibration

An animal feed sample was prepared by adding 1 g of feed material comprising lucerne and partially fermented timothy grass (Fiber Ezy; Fiber Fresh, Reporoa, New Zealand) to 10 mL of PBS and mixing by vortexing for 1 min. The feed sample was incubated for 1 h at room temperature and filtered through a 100 µm cell strainer (Corning, Hamburg, Germany) prior to spectral flow cytometry analysis.

Rumen content samples collected from sheep (*n* = 10) or cattle (*n* = 23) were centrifuged at 13,000 *g* for 20 min at room temperature. After centrifugation, the supernatant was transferred into a clean tube, and the material was again centrifuged. This process of centrifugation was repeated one more time, and then the clarified supernatant was transferred into a clean tube and used as rumen fluid (as distinct from rumen contents) for flow cytometry analysis.

### Isolation of genomic DNA for qPCR

Total DNA was extracted from samples of pure methanogen cultures and fresh rumen content samples as described previously (46). Briefly, cells were disrupted using a combination of bead-beating (FastPrep FP120; Qbiogene, Carlsbad, CA, USA) at 6.5 ms^−1^ for 45 s and a 20% SDS solution in Tris buffer (200 mM NaCl, 200 mM Tris, 20 mM EDTA, pH 8). Samples were then centrifuged at 14,000 RPM for 20 min at 4°C, with supernatants kept on ice. DNA was extracted from the supernatants using a QIAquick PCR purification kit (QIAGEN, Germantown, MD, USA) and stored at −20°C for qPCR.

### Quantitative PCR

Methanogens from fresh and filtered rumen content samples were quantified using a Rotor-Gene 6000 real-time rotary analyzer (Corbett Life Science, Concorde, NSW, Australia) with amplicon detection using SYBR Green I fluorescence (Light Cycler Fast-Start DNA Master SYBR Green I Kit; Roche, Auckland, New Zealand). The extracted DNA was used as a template for PCR to amplify the archaeal 16S rRNA gene using forward primer Ar915af targeting 16S rRNA gene nucleotide positions 915-934 (5′-AGGAATTGGCGGGGGAGCAC-3′) (47) and reverse primer Ar1386R targeting positions 1,386–1,403 (5′- GCGGTGTGTGCAAGGAGC-3′) (48). Nucleotide positions were based on published reference sequences (49). Plasmids containing archaeal 16S rRNA gene inserts were constructed, quantified with the Quant-iT dsDNA BR Assay Kit on a Qubit fluorometer (Invitrogen, Carlsbad, CA, USA), and diluted 10-fold in series to produce five standards ranging from 2 × 10^3^ to 2 × 10^7^ copies per reaction, each in duplicate for use in the qPCR. Each reaction (20 μL) in PCR tubes contained 10 µL Light Cycler Mix, 2µL of primers mix (5 µM each), 6 µL of nuclease-free water, and 2 µL of standard or DNA template. The thermal protocol for qPCR amplification and detection was 10 min of initial denaturation (95°C), followed by 40 amplification cycles (10 s at 95°C; 5 s at 59°C; 10 s at 72°C). Following each run, melting curves between 72°C and 95°C were evaluated to confirm the absence of non-specific signals.

### F_420_ reduction

Samples were prepared from each cultured methanogen isolate (M1, AbM4, SM9, D5, and 229/11), from a mixture of equal amounts of these five isolates (Mix5), and from a fresh rumen content sample obtained from an untreated control animal, as described above. A solution of 1 M sodium borohydride (NaBH_4_; Sigma, Auckland, New Zealand) was added to an ice-cold solution of 1 N NaOH. To reduce F_420_ autofluorescence of methanogens, 50 µL of 1 M NaBH_4_ solution was added to a 1 mL suspension of 4.3 × 10^7^ cells (based on microscopy counts) in PBS of each single methanogen isolate or total cells in Mix5 or to 1 mL of 50-fold diluted rumen content samples from animals. Samples were incubated at room temperature for 1 h with gentle rotation. The effect of NaBH_4_ on the intensity of methanogen F_420_ fluorescence was determined by flow cytometry and fluorescence microscopy.

### Membrane integrity assay

To test the membrane integrity of methanogen cells after treatment with NaBH_4_, samples of M1 cells were prepared based on the instructions given in the Live/Dead *Bac*Light bacterial viability kit (ThermoFisher Scientific, Willows, CA, USA). M1 cell cultures were harvested by centrifugation at 5,000 *g* for 5 min. The resulting cell pellet was washed three times in PBS to remove any culture medium. Cells were resuspended in PBS. Washed cells were used as the live M1 sample at a concentration of 1 × 10^6^ cells/mL. To prepare dead cells, M1 samples containing 1 × 10^6^ cells/mL were treated with 70% isopropanol followed by incubation at room temperature for 1 h with periodic mixing. Samples were harvested by centrifugation at 5,000 *g* for 5 min at room temperature, and the resultant pellets were resuspended in 1 mL of PBS prior to repeating the centrifugation step with a final resuspension in 1 mL of PBS. M1 cells (1 × 10^6^ cells/mL) treated with NaBH_4_ were prepared as mentioned above, then harvested by centrifugation and resuspended in 1 mL of PBS. A working solution of SYTO9 and propidium iodide was prepared by mixing equal amounts of both reagents from the Live/Dead *Bac*Light bacterial viability kit. Each M1 sample (live, dead, and NaBH_4_ treated) was stained by adding 3 µL of combined SYTO9 and propidium iodide reagent. Samples were thoroughly mixed by pipetting and incubated at room temperature in the dark for 15 min. After staining, samples were analyzed by flow cytometry using excitation/emission spectrum of 488/500 nm for SYTO9 stain and 488/635 nm for propidium iodide. Control samples were prepared in parallel but omitting treatment with NaBH_4_ or isopropanol.

### Fluorescence microscopy

*M. ruminantium* M1 and rumen content samples were photographed using a Leica DM2500 fluorescence microscope with a 100× objective (Leica, Wetzlar, Germany) with a UV filter system D (excitation BP 355–425 nm, 455 nm dichromatic mirror, and 470 nm long pass-suppression filter). F_420_ autofluorescence of cultured methanogen cells (Mix5) before and after treatment with NaBH_4_ was visualized and photographed using an Olympus BX61 fluorescence microscope with a 100× objective (Olympus, Tokyo, Japan) with a UV filter system (excitation BP 330–385 nm, 400 nm dichromatic mirror, and 420 nm emission filter).

### Quantification of *M. ruminantium* M1 cells using microscopy

M1 cells were harvested from fresh M1 cultures and washed in PBS as described above. A measured volume of the washed cell suspension was then loaded into a Petroff-Hausser counting chamber (Weber Scientific International, Teddington, United Kingdom). Cells were counted under an inverted light microscope (Leitz, Wetzlar, Germany) at 400× magnification. The average number of cells per grid square was recorded and used, together with the chamber volume and dilution factor, to calculate the concentration of cells in the original suspension. Cell counts were performed in triplicate. Predicted cell counts for each subsequent 10-fold dilution were derived by applying the appropriate dilution factor to this calculated concentration.

### Fixation of methanogens and determination of rumen sample stability

An aliquot of 1 × 10^7^ washed M1 cells in 100 µL PBS was treated with 100 µL 4% (vol/vol) paraformaldehyde. Samples were mixed thoroughly before incubation at room temperature for 30 min with slight agitation. Similarly, 50-fold diluted fresh rumen content samples in PBS were fixed by the addition of paraformaldehyde to a final concentration of 2% (vol/vol), followed by incubation at room temperature for 30 min with slight agitation. Samples of fixed M1 cells and rumen content were thoroughly washed with PBS, and the resulting pellet was reconstituted in 1 mL PBS. These samples were then analyzed by spectral flow cytometry to detect the methanogen-specific autofluorescence of F_420_ and compared with an unfixed sample. Unfixed samples stored at 4°C were also analyzed daily for up to 3 days to determine the stability of the autofluorescence.

### Flow cytometry

Flow cytometric analysis of methanogens in cultures and rumen content was performed using a 3-laser Cytek Aurora flow cytometer (Cytek Biosciences, Fremont, CA, USA). The configurations used are shown in Table S3. Quality check procedures were performed on the instrument daily and before analyzing samples. Spectral unmixing was performed with SpectroFlo software (Cytek Biosciences) using reference controls as described and optimized in Fig. S1 to S3. The staining index (SI) equation was used as a metric for optimal signal resolution (although no stains were employed). The SI was calculated as the difference between the mean fluorescence intensity of the positive population and the negative population, divided by twice the robust standard deviation of the negative population. Figures were prepared, and data were analyzed using SpectroFlo software or Flowjo v10 software for both Windows and Mac (both Becton, Dickinson and Company, Ashland, OR, USA).

### Statistical analysis

To evaluate the precision of M1 spectral cytometry counts, statistical error (SE) was calculated for the mean of the total methanogen count in the gate (prior to adjustment for cells/mL) of each dilution. The error was expressed as a percentage to indicate variability relative to the mean. This approach allowed us to identify the dilution level that provided the most reliable measurement. The lowest dilution with an SE of less than 5% was considered acceptable for further analysis.

## RESULTS

### Unique F_420_ signal distinguishes autofluorescent methanogens from other particles in rumen content samples

**Fig 1:**
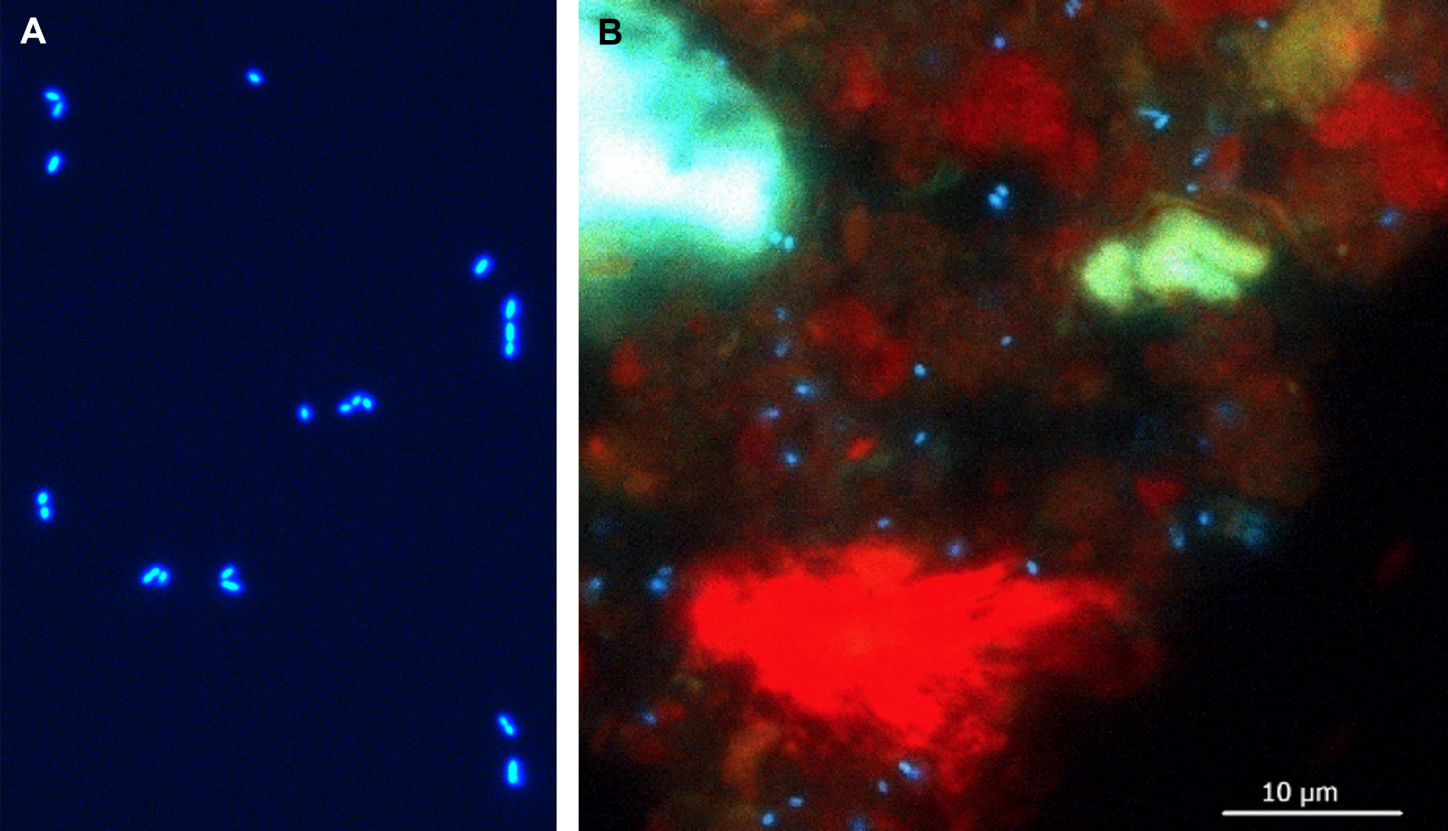
(**A**) Cultured methanogen *M. ruminantium* M1 and (**B**) rumen content imaged by fluorescence microscopy, showing methanogens expressing F_420_ co-enzyme fluorescing as small blue-emitting particles, alongside the plant-derived larger fluorescent material emitting in green and red wavelengths. Samples were observed using a fluorescence microscope with a 100× objective with a UV filter system D (excitation BP 355–425 nm, 455 nm dichromatic mirror, and 470 nm long pass-suppression filter). Both panels are at the same magnification, and the scale bar applies to both panels.

**Fig 2:**
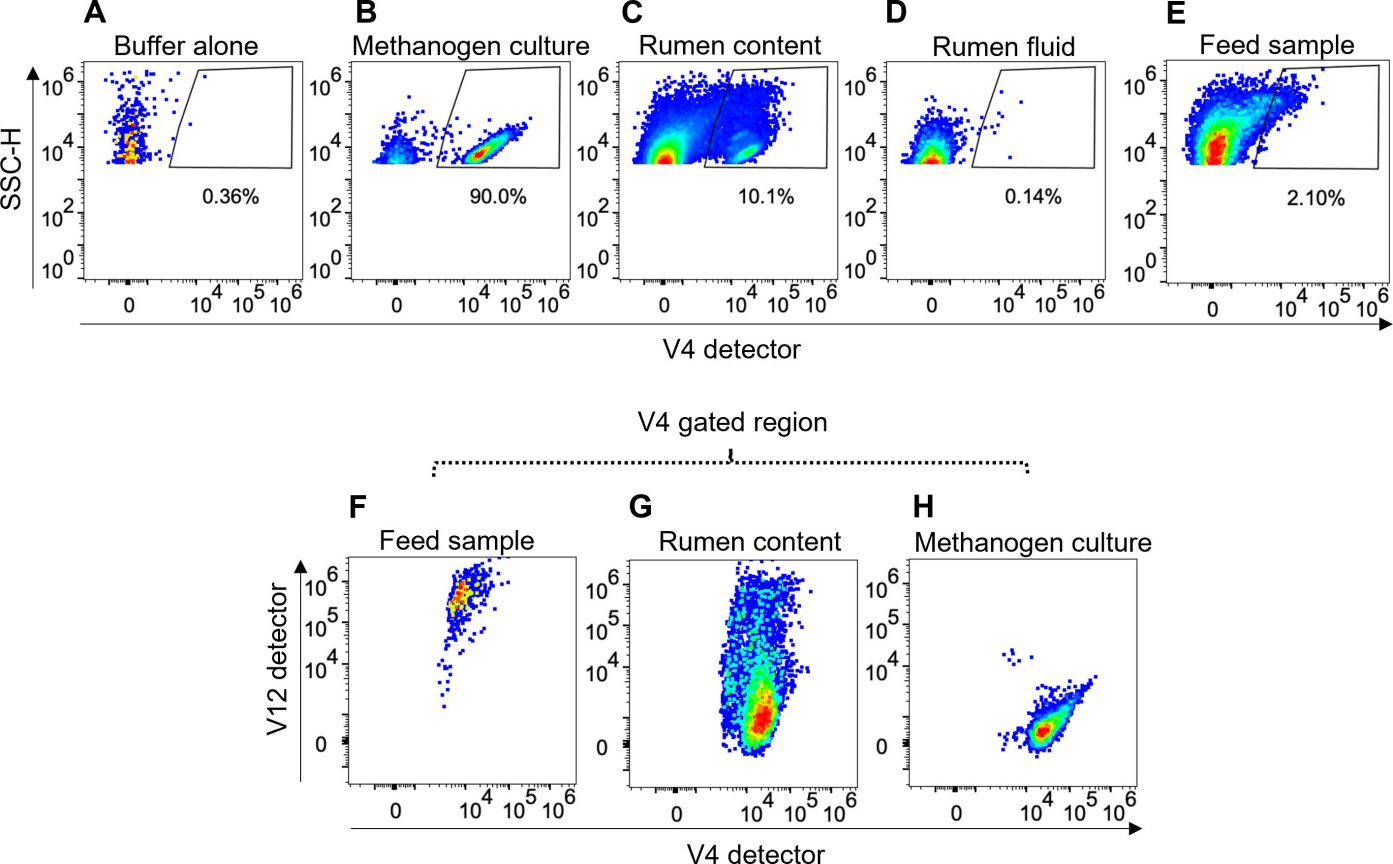
Cultured methanogens (*M. ruminantium* M1) in buffer and native rumen methanogens in a rumen content sample were measured using conventional cytometry (single detectors) with F_420_ autofluorescence detected in the V4 detector (405 nm laser with bandpass filter BP 473/15 nm). Percentage values represent the proportion of particles within the gate on each plot. (**A**) Buffer alone contained negligible particles with F_420_-like autofluorescence. (**B**) Cultured methanogens were predominantly comprised of F_420_ autofluorescent particles detected in V4. (**C**) Autofluorescence in V4 was also detected in a rumen content sample, with V4 autofluorescent particles showing a range of sizes, including larger particles with increased SSC-H. (**D**) No methanogens were detected in rumen fluid after suspended material had been removed by centrifugation. (**E**) A ryegrass feed sample also contained particles with bright V4 autofluorescence and a higher SSC-H signal. (**F–H**) Some particles identified by the V4 gate in panels **A–E** were autofluorescent in V12 (405 nm laser, BP 692/28) in both (**F**) feed and (**G**) rumen content samples, but not in (**H**) cultured methanogens, suggesting autofluorescent particles identified in V12 and V4 were likely not methanogens. Data for methanogen cultures and rumen content are representative of three independent experiments; the feed sample is representative of two independent experiments.

Cultured methanogens of the order *Methanobacteriales* fluoresced brightly when observed using a microscope under UV excitation, based on their intrinsic fluorescence due to F_420_ (Fig. 1A). Rumen samples also contained putative F_420_-containing methanogens that fluoresced as small blue-emitting particles the same size as the cultured methanogens, alongside the larger, plant-derived fluorescent material emitting in green and red wavelengths (Fig. 1B). For methanogen quantification, it is important to distinguish the methanogen population from the plant material present in the rumen content. To establish the method for methanogen detection and quantification using flow cytometry, we first used pure cultures of *M. ruminantium* M1. M1 cells fluoresced brightly with flow cytometry violet laser excitation, which was absent in the buffer-only control (Fig. 2A), as expected (25, 26). A discrete, relatively uniform population of events was observable using 473/15 bandpass filter (V4 detector), accounting for the majority of particles present in the cultured M1 sample (Fig. 2B). Using side scatter (SSC) as a measure of proportionate size and complexity, the small size of these organisms is indicated by the low SSC signal. Instrument settings were adjusted to capture signals from these samples as described in Text S1. SSC is more sensitive than forward scatter (FSC) for microbial size discrimination (50).

Rumen content samples were prepared by collection from cattle rumen and filtering through a 100 µm cell strainer before diluting in PBS (1:500). A population of methanogens was identified in the V4 detector with similar characteristics to the cultured methanogens, but other data in this sample with a strong V4 signal displayed higher SSC than cultured methanogens, indicating larger size particles present in the rumen content (Fig. 2C). No population of V4 positive (V4+) particles was detected in the rumen fluid that was centrifuged multiple times, suggesting that the methanogens were contained in the pellet (Fig. 2D).

Constituents other than methanogens could explain the larger particles in rumen content samples that displayed methanogen-like V4 fluorescence in Fig. 2D. Suspecting that these might be derived from feed (plant) material, a feed sample was prepared and measured for comparison. This showed particles of larger SSC that fell in the V4+ region where methanogens were expected (Fig. 2E). From this, we postulated that the larger particles in the putative methanogen region in Fig. 2E were derived from the diet of the ruminant.

To explore this further, we considered that a distinct autofluorescence profile for these larger rumen content particles could be possible. Indeed, the V12 detector (692/28 bandpass filter) gave a strong signal from both the feed sample (Fig. 2F) and rumen content sample (Fig. 2G), while the pure culture of methanogens showed no signal in this detector (Fig. 2H). This suggests that methanogens could easily be distinguished from large autofluorescence rumen particles based on their distinct spectral profile.

**Fig 3:**
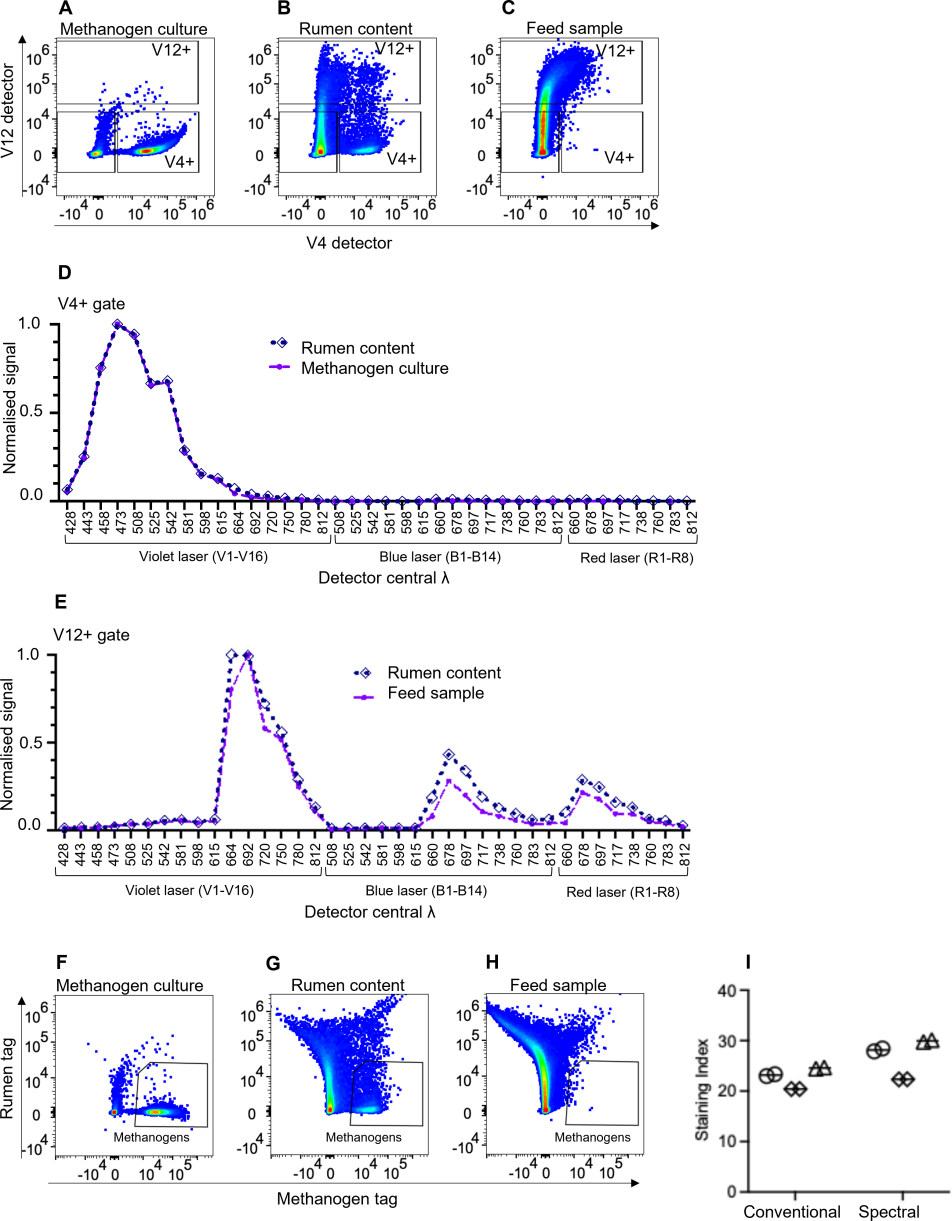
Spectral flow cytometry delineated methanogens from plant-derived particles in rumen content samples. (**A–C**) Conventional cytometry using V4 and V12 detectors defined regions of interest from (**A**) cultured methanogens, (**B**) rumen content, and (**C**) a feed sample. Samples in panels **A–C** were pre-gated using FSC-H vs SSC-H to exclude very large signals from rumen content and were time gated for signal stability. (**D and E**) Normalized spectral profiles of regions of interest from panels **A–C** are shown. (**D**) Normalized spectral profiles for the V4+ gate in both cultured methanogens and rumen content samples showed identical spectral profiles, indicating that these particles in rumen content were methanogens. (**E**) Normalized spectral profiles for V12+ from rumen content and a feed sample show a strikingly similar spectral profile that is distinct from the methanogen profile, suggesting such particles in rumen content were plant derived. (**F–I**) Using spectral flow cytometry, data were unmixed with spectral profiles as shown in panels **D** and **E** using V12+ from the rumen content sample and V4+ from the methanogen sample. This identified methanogens in (**F**) cultured methanogens and (**G**) rumen content and showed an absence of methanogens in (**H**) the feed sample. (**I**) A staining index calculation confirmed that the resolution of methanogens from background is superior with spectral data compared to conventional data. Data in panels **A–H** for methanogen cultures and rumen content are representative of three independent experiments, and the feed sample is representative of two independent experiments. Data in panel **I** are from three individual sheep rumen content samples, indicated by different symbols, each analyzed in duplicate.

Full-spectrum profiling of methanogens from pure methanogen cultures and rumen content samples (V4+ as shown in Fig. 3A and B, respectively, which were absent in the feed sample in Fig. 3C) revealed an identical spectral profile. This strongly suggested that these V4+ but V12-dim particles in rumen content samples were native rumen content methanogens (Fig. 3D).

Examining the full spectrum profile of these unknown particles from rumen content (defined as V12+ signal as shown in Fig. 3B) revealed that they had a distinct spectral profile that had close resemblance to spectra obtained from the same analysis of the feed sample (Fig. 3E). This suggested that these populations are both likely to originate from plant material.

The spectral profile of V12-positive particles in rumen content was in agreement with the microscopic image of a rumen content sample showing small blue-emitting methanogens and larger fluorescent material emitting in green and red wavelengths (Fig. 1). The red-fluorescent material is likely the plant-derived material identified in the V12 detector in Fig. 3E.

Having identified unique fluorescent profiles for the key particles detected in rumen contents, we proposed that a spectral unmixing approach could be used to create unified fluorescent tags for these two distinct components of the rumen content milieu. Using cultured methanogens as a reference for the methanogen fluorescent tag during spectral unmixing, and “All V12 bright” particles from rumen content as a reference for the rumen content fluorescent tag (Fig. S1C), we obtained unmixed spectral data. These data produced a F_420_-containing methanogen-specific tag (Fig. 3D) with improved sensitivity for detecting methanogens in both methanogen cultures (Fig. 3F) and rumen content samples (Fig. 3G), compared to single-detector (raw) data. Additionally, no methanogens were detected in the feed sample (Fig. 3H).

Different populations in rumen content samples were selected and analyzed for use as the reference control for spectral unmixing of the rumen content fluorescent tag. A staining index to assess the resolution of the methanogen signal was calculated for each unmixing approach in rumen content samples to determine the optimal population for use as a reference control. Details of these selected population employed as reference controls for the various unmixing approaches are given in Fig. S1, and the staining indices are given in Fig. S2. The staining index is a commonly used metric to assess signal resolution and was computed on the autofluorescence of the methanogens present in the rumen content samples. Nevertheless, no staining with dyes was involved in the preparation of these samples. The optimal reference control was determined by (i) comparing the staining indices produced by the different approaches, (ii) comparing the spread of the rumen contents tag into the methanogens tag using the coefficient of variation (%CV; Fig. S3), and (iii) the ease of selecting reference control material between experiments. Based on the above factors, the rumen content population “All V12 bright” was selected as the reference control for the rumen content tag for unmixing (Fig. S3).

Methanogen cultures used in this study were grown in BY^+^ medium, which contained 30% of rumen fluid. Although cultured methanogens were washed extensively before analyses, it is possible that some non-methanogen counts may be due to particles contained in the media used to culture the methanogens. In support of this interpretation, spectral flow cytometry analysis of a medium-only sample showed a spectrum similar to a rumen content sample treated with NaBH_4_ to quench methanogen autofluorescence (Fig. S4).

Our data show that a spectral flow cytometry unmixing approach increases the resolution of the autofluorescent methanogen population in rumen content samples by facilitating the exclusion of signals from feed material in the rumen content and removing ambiguity caused by the presence of particles with higher SSC.

### Validation of the gated autofluorescent methanogen population

**Fig 4:**
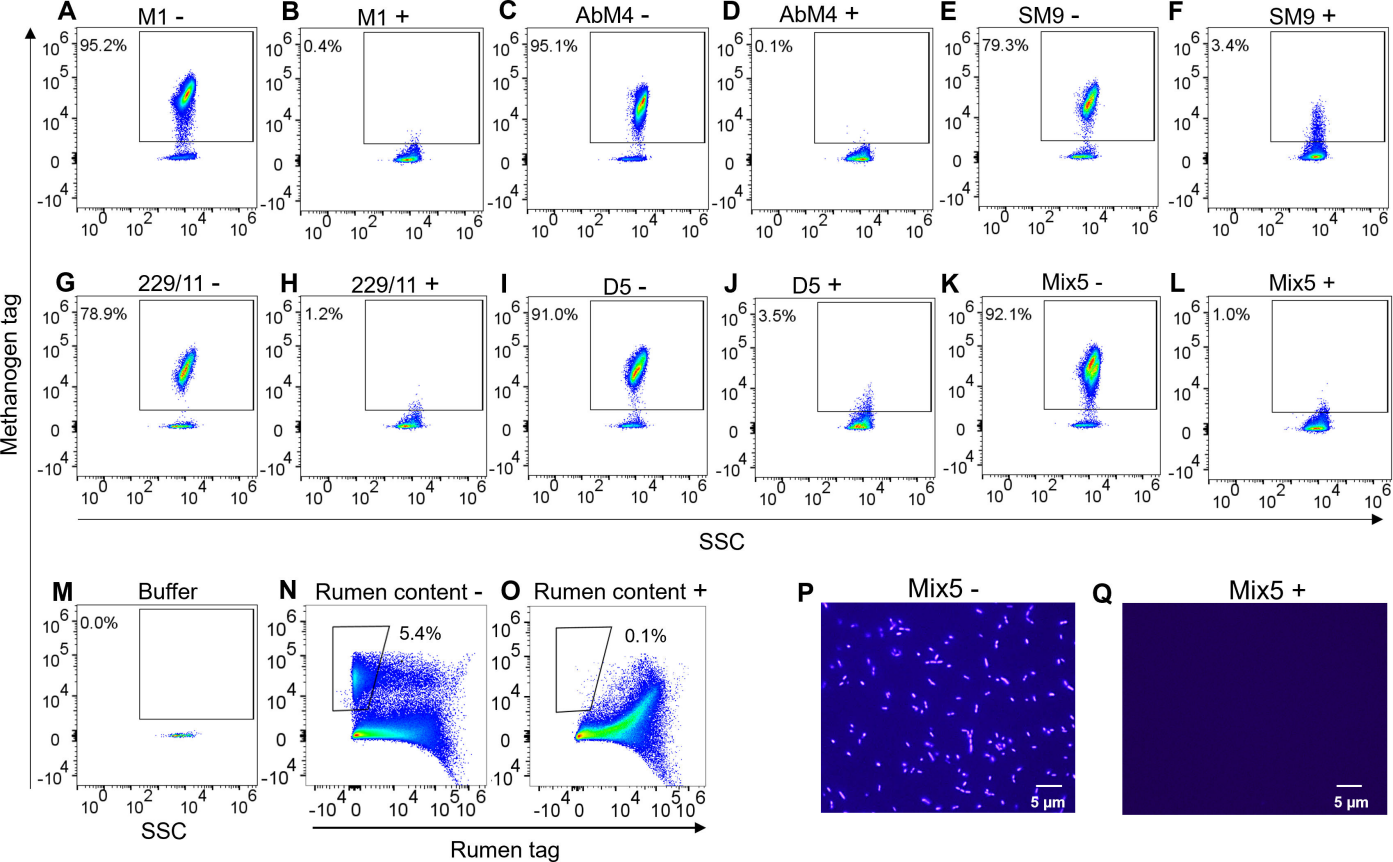
Methanogen autofluorescence was detected using spectral flow cytometry in five distinct methanogen isolates and ablated in all five, as well as in rumen content, by NaBH_4_, a known inhibitor of F_420_ autofluorescence. Following spectral unmixing, the methanogen gate was defined based on the buffer-only control and with reference to the clear methanogen signal in untreated methanogen cultures. Percentage values represent the proportion of particles within the methanogen gate from the overall population. The samples without treatment are represented by “–,” while those treated with NaBH_4_ chemical are indicated by “+.” (**A**) *M. ruminantium* M1 before and (**B**) after NaBH_4_ treatment, (**C**) *M. boviskoreani* AbM4 before and (**D**) after NaBH_4_ treatment, (**E**) *M. millerae* SM9 before and (**F**) after NaBH_4_ treatment, (**G**) *M. olleyae* 229/11 before and (**H**) after NaBH_4_ treatment, (**I**) *Methanobrevibacter* sp. D5 before and (**J**) after NaBH_4_ treatment, (**K**) Mix5 (five cultures combined) sample before and (**L**) after NaBH_4_ treatment, (**M**) buffer only control, and (**N**) fresh rumen content sample before and (**O**) after NaBH_4_ treatment. Fluorescence microscopic images showing (**P**) cultured Mix5 methanogens, which naturally fluoresce under UV light due to F_420_ and emit a blue fluorescence with an emission at 420 nm, and (**Q**) cultured Mix5 methanogens after treatment with NaBH_4_.

The authenticity of the methanogen population that was identified based on intrinsic F_420_ autofluorescence was further confirmed by treating individual isolates of cultured methanogens of the genus *Methanobrevibacter* (isolates M1, AbM4, SM9, D5, and 229/11), a mixture of these five isolates (Mix5), and a fresh bovine rumen content sample with sodium borohydride (NaBH_4_). As expected, treatment with NaBH_4_ reduced F_420_ autofluorescence, resulting in an almost total loss of the methanogen signal (Fig. 4A through M). Depending on the isolate of cultured methanogen, particles detected in the methanogen gate were reduced from 87.9% to 99.3% to between 0.1% and 5.2% of the total after treatment with NaBH_4_ (Fig. 4B, D, F, H, J and L). Treatment of the rumen content samples with NaBH_4_ caused a reduction of particles with methanogen autofluorescence from 5.1% of the total particles to 0.1% (Fig. 4N and O). Reduction of methanogen autofluorescence by treatment of cells with NaBH_4_ was additionally shown by observing methanogens (Mix5) with fluorescence microscopy before and after treatment with NaBH_4_ (Fig. 4P and Q).

To confirm that NaBH_4_ treatment did not reduce F_420_ fluorescence by damaging cell membrane integrity, leading to cell death and fluorescence loss, NaBH_4_-treated M1 samples were stained simultaneously with SYTO9 and propidium iodide to distinguish live and dead cells, respectively. No effect on cell membrane integrity was observed (Fig. S3), suggesting that NaBH_4_ treatment affected F_420_ autofluorescence specifically, without causing cell membrane damage.

**Fig 5:**
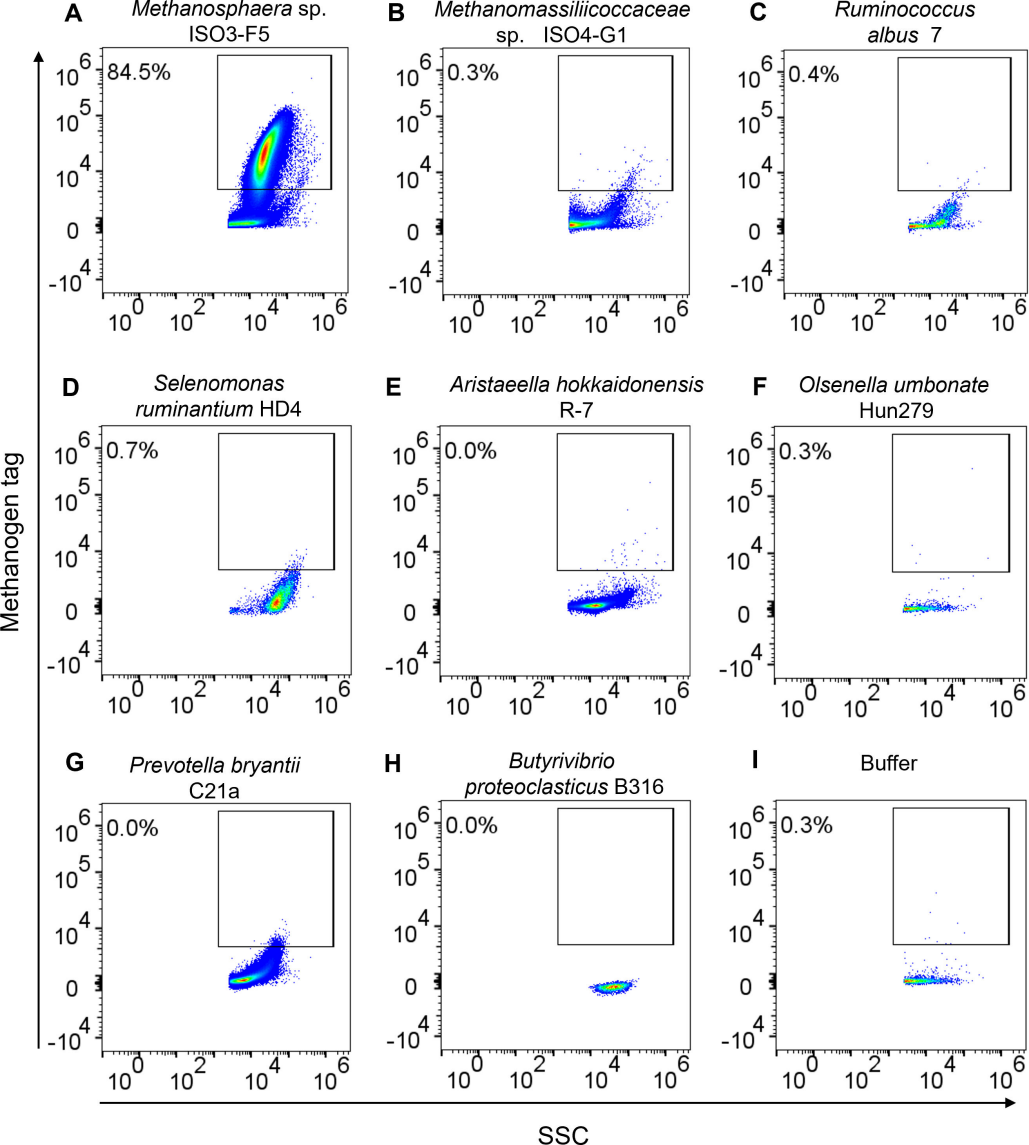
Methanogen autofluorescence in pure cultures of other representatives of the rumen microbial community is of low abundance and low intensity. Following spectral unmixing, the methanogen and buffer gates were defined based on the buffer-only control and with reference to the clear methanogen signal from the methanogen culture. Percentage values represent the proportion of particles within the methanogen-bright gate from the overall population. Cultures lacking F_420_ fluorescence appear in the buffer gate. Samples used in this analysis were (**A**) *Methanosphaera* sp. ISO3-F5, (**B**) *Methanomassiliicoccaceae* isolate ISO4-G1, (**C**) *R. albus* 7, (**D**) *S. ruminantium* HD4, (**E**) *A. hokkaidonensis* R-7, (**F**) *O. umbonata* Hun279, (**G**) *P. bryantii* C21a, (**H**) *B. proteoclasticus* B316, and (**I**) buffer only.

We assessed the presence of the methanogen signal in other microbial species found in rumen content. *Methanosphaera* sp. ISO3-F5 was detected within the methanogen region previously defined with *Methanobrevibacter* spp., as expected from its close relationship to the genus *Methanobrevibacter* and known autofluorescence (Fig. 5A). In contrast, cells of the non-fluorescent methanogen isolate ISO4-G1, a member of the *Methanomassiliicoccales* (34), were not detected (Fig. 5B). Similarly, non-fluorescent rumen bacterial isolates *R. albus* 7, *S. ruminantium* HD4, *A. hokkaidonensis* R-7, *O. umbonata* Hun279, *Prevotella bryantii* C21a, and *B. proteoclasticus* B316 were not detected in the methanogen region/gate (Fig. 5C through H). These results indicated that identification of methanogens based on their F_420_ autofluorescence using spectral cytometry would discriminate the population of autofluorescent methanogens from non-autofluorescent members of the microbial community in rumen content samples.

### Quantitation of cultured autofluorescent methanogens in buffer

The spectral flow cytometry protocol used to detect methanogens in buffer and rumen content was optimized to count the number of methanogens present in buffer samples containing *M. ruminantium* M1. A total count, rather than a percentage, is often required when assessing the efficacy of anti-methanogenic interventions, as this gives an absolute read-out of methanogen reduction. Many cytometers equipped with volumetric counting can analyze count per milliliter automatically without the addition of counting beads, storing the count as part of the flow cytometry standard file, and such a system was used here.

**Fig 6:**
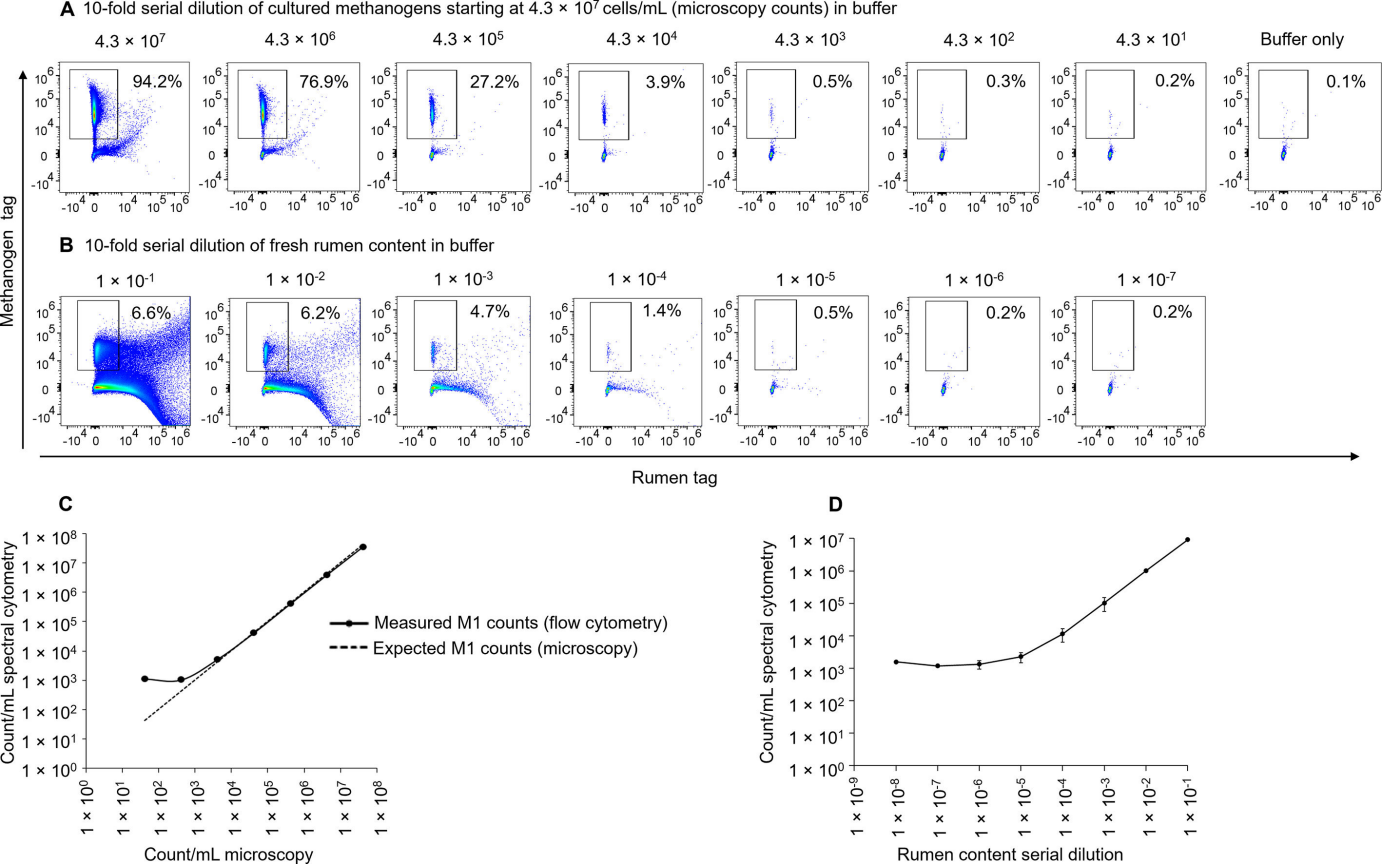
Absolute methanogen counts by spectral flow cytometry from pure cultures and from rumen content were titratable over a broad concentration range and corresponded with microscopy counts for pure cultures. (**A**) Cultured *M. ruminantium* M1 cells were washed and serially diluted in buffer before analysis, from 4.3 × 10^7^ to 4.3 × 10^1^ cells/mL with the final plot showing buffer alone. (**B**) Fresh rumen content samples were collected, filtered, and diluted 50-fold. This 50-fold diluted rumen contents were used to make 10-fold serial dilutions in buffer out to a 5 × 10^8^-fold dilution. Percentage values represent the proportion of particles within the methanogen gate from the overall population. (**C**) Absolute counts from triplicate dilutions of pure cultures shown in panel **A** are plotted compared to the expected number of methanogens determined by microscopy. The samples were analyzed by microscopy using a Petroff-Hausser counting chamber under inverted light microscope at 400× magnification and using spectral flow cytometry. The expected relationship between spectral flow cytometry data and microscopy data is shown by the dotted line. (**D**) Absolute counts from triplicate dilutions of rumen content data shown in panel **B** are plotted compared to the dilution.

A 10-fold dilution series of cultured M1 cells in buffer was prepared over a wide range of concentrations starting at 4.3 × 10^7^ cells/mL and used to count methanogens (Fig. 6A and C). Spectral cytometry results were cross-referenced to light microscopy counts for the highest concentration and to predicted counts (based on serial dilution of the highest concentration) for other data points. SE of the cytometry count (i.e., SE of the total count of methanogens in the gate prior to cells per milliliter adjustment) was less than 5% for samples with concentrations of 4.05 × 10^4^/mL and above, making this the lower limit of accurate detection (Table S4). At lower cell concentrations, the SE exceeded 5%, substantially due to low total count of methanogens in the gate. Values below this point were deemed out of range.

### Quantification of native autofluorescent methanogen populations in fresh rumen content samples

Native methanogens in fresh rumen content samples were counted using the spectral flow cytometry assay described so far (Fig. 6B). To assess the limit of detection, we used a 10-fold dilution series in buffer prepared from an initial 50-fold diluted and filtered rumen content sample. Graphical representation of native methanogen counts of three technical replicates of a fresh rumen content sample from an animal not treated with a methanogen inhibitor showed a linear titratable methanogen count (Fig. 6D). A dilution of rumen content between 1:100 and 1:5,000 gave more accurate methanogen counts than the lowest dilution range (1:50,000 to 1:50,000,000). Methanogens present in rumen content samples at levels below 10^3^ counts/mL were not accurately countable, and thus, again, 10^4^ counts/mL is the lowest limit of our current protocol for methanogen quantification (Fig. 6D).

### Sample stability

**Fig 7:**
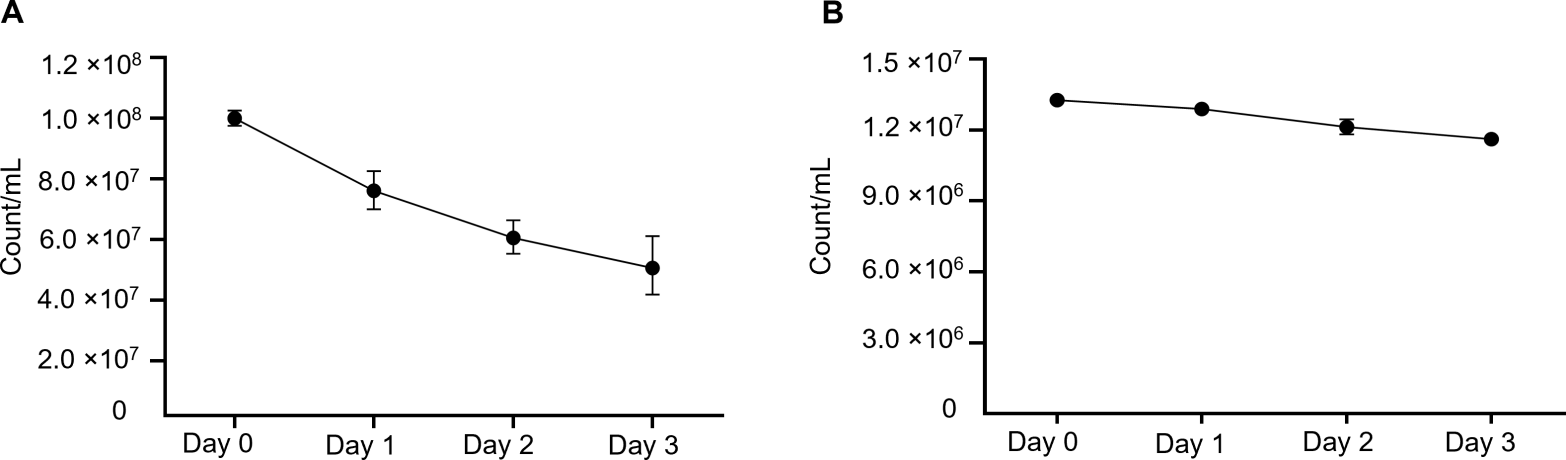
Native rumen methanogens in rumen content were more stable at 4°C than cultured methanogens. Samples of (**A**) cultured *M. ruminantium* M1 stored in buffer and (**B**) fresh rumen content samples diluted 1:50 in buffer and stored at 4°C for up to 3 days. Samples were analyzed using spectral flow cytometry to determine methanogen counts as described. Error bars represent the standard deviation of three technical replicates.

To develop a reliable protocol to store samples that causes minimal technical biases to the recorded fluorescence intensity and absolute count of autofluorescent methanogens in rumen content samples, we tested the effect of storing cells of *M. ruminantium* M1 or rumen content samples. Two storage protocols were tested: (i) storage in PBS buffer at 4°C, and (ii) fixation of *M. ruminantium* M1 cells in 2% (vol/vol) paraformaldehyde for 30 min, followed by storage at 4°C. The samples were analyzed by spectral flow cytometry daily over a 3-day period to determine the number of methanogens able to be detected. The counts of autofluorescent methanogens reduced over time in the un-fixed cultured M1 samples stored at 4°C (Fig. 7A), with approximately 20% of the counts lost after 1 day, 30% after 2 days, and 40% after 3 days of storage. In comparison, the decline of detectable unfixed methanogens over time in rumen content during storage was much less, being reduced to 13% over 3 days storage at 4°C (Fig. 7B). Fixing *M. ruminantium* M1 cells and rumen content by treatment with 2% paraformaldehyde for 30 min resulted in an immediate reduction in counts, with approximately 35% reduction observed in M1 cells and 14% in rumen content samples (Fig. S6). The average per cell autofluorescence was reduced in intensity compared to unfixed methanogens, but the detection of these cells was still easily achieved (data not shown).

### Effect of a methanogen inhibitor on native autofluorescent methanogen counts in rumen content samples from cattle

**Fig 8:**
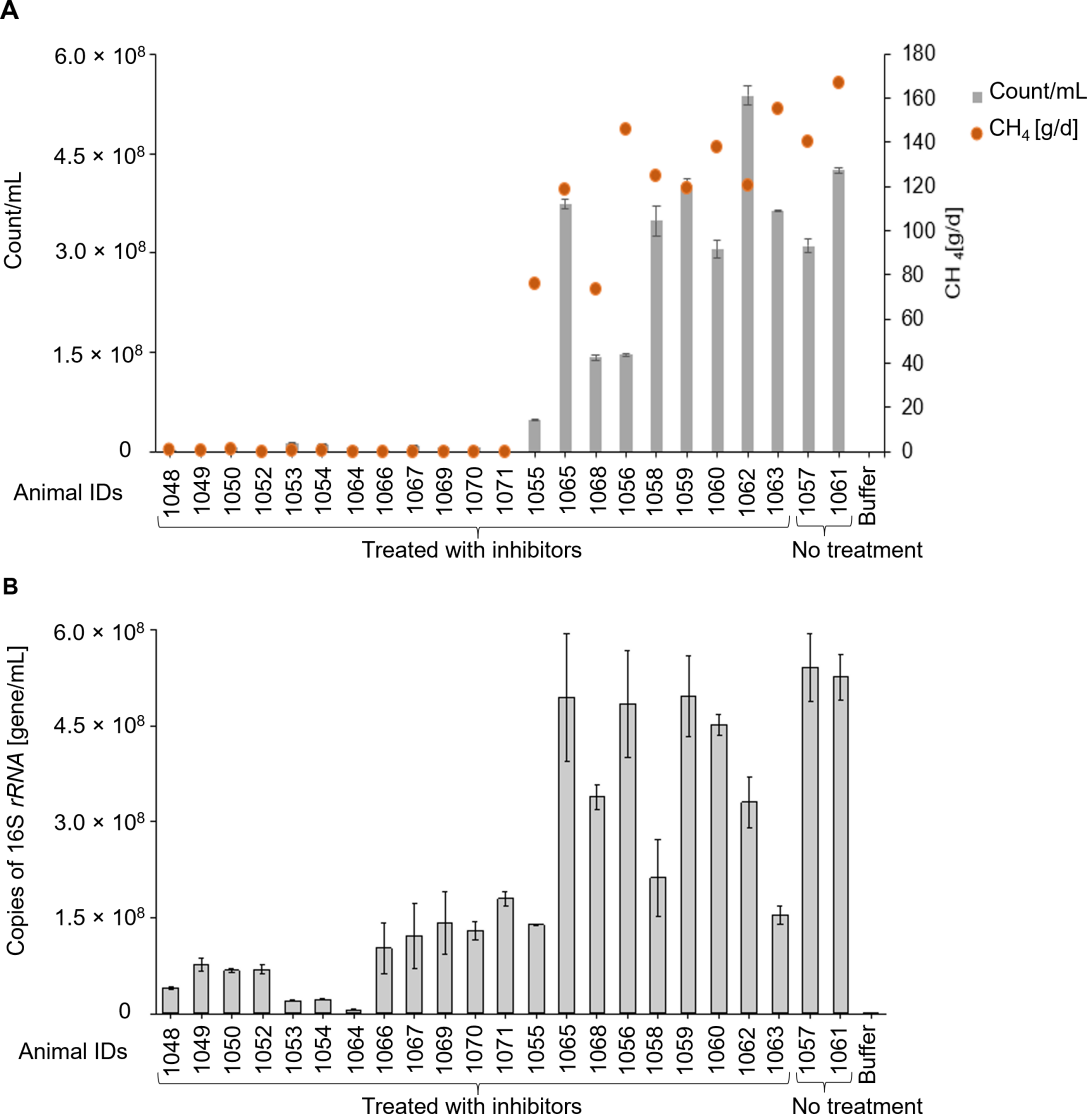
Native F_420_-expressing methanogens in rumen content samples from cattle treated with a methanogen inhibitor. (**A**) Native methanogens counted using spectral flow cytometry in fresh rumen content samples from individual cattle (*n* = 23) were assessed and plotted with methane emission data collected at the same time from the same animal. (**B**) Genomic DNA was isolated from the frozen rumen content samples, and methanogen numbers were quantified by qPCR using primers to amplify the 16S *rRNA* gene. Data are presented as means of (±SE) three technical replicates. Animal IDs 1,057 and 1,061 served as controls and were not treated with inhibitors.

Rumen samples were collected from cattle being treated with a methanogen inhibitor, along with measures of methane emissions from these cattle. The number of autofluorescent methanogens in these samples was analyzed using spectral flow cytometry. The methanogen counts ranged from 9.95 × 10^5^ cells/mL in samples from animals with low methane emissions, intermediate in animals with intermediate methane inhibition, and up to 5.38 × 10^8^ cells/mL in samples from animals with minimal inhibitory effects due to being administered a lower or an ineffective dose of the inhibitor or from the control animals that were not administered inhibitor (Fig. 8A). These counts were further confirmed by performing qPCR on the same rumen content samples, with overall good agreement between methanogen counts obtained by spectral flow cytometry and methanogen concentrations determined by measuring copies of 16S *rRNA* genes (Fig. 8B).

### Effect of a methanogen inhibitor on native methanogen counts in rumen content from sheep

**Fig 9:**
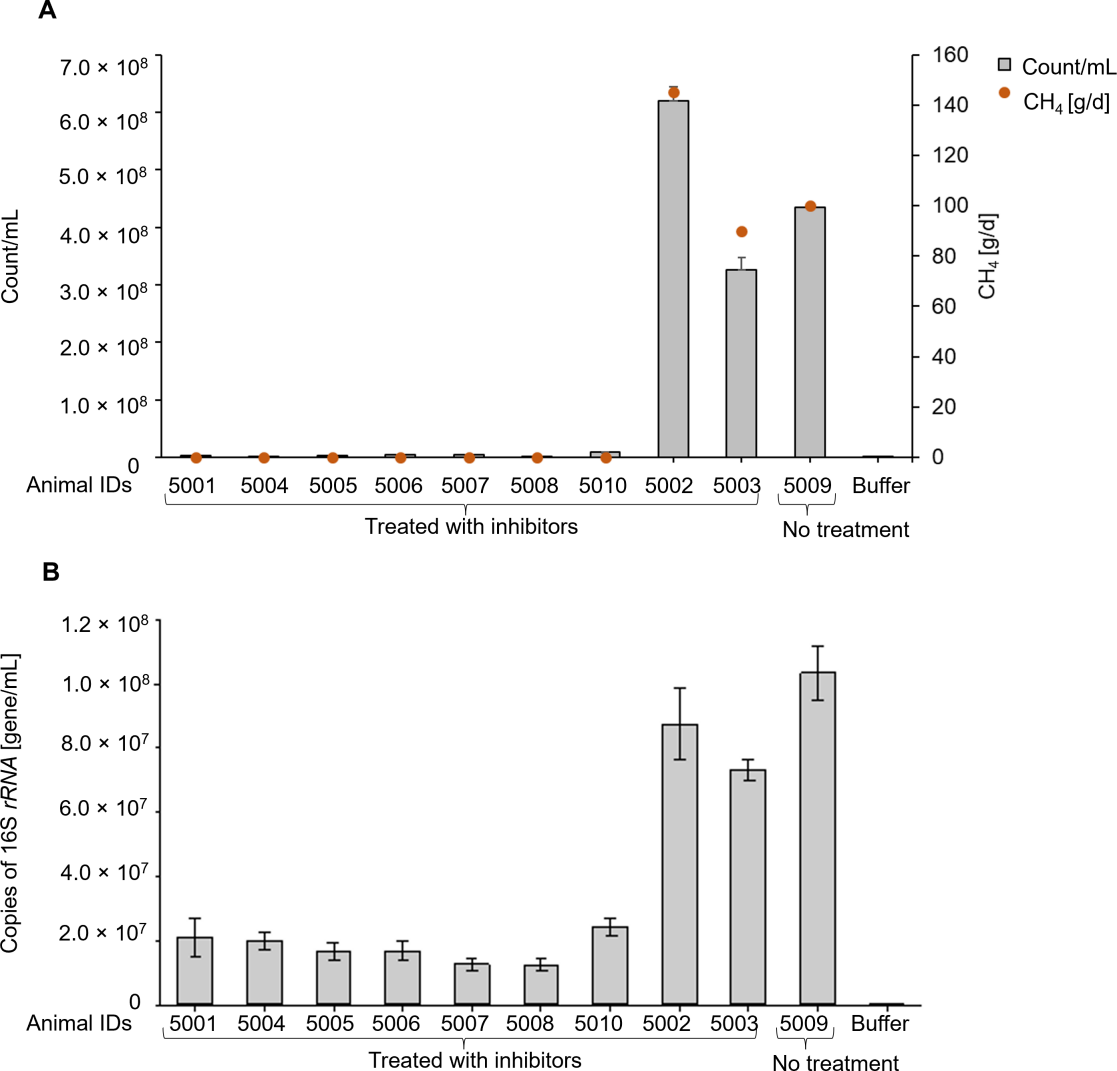
Native F_420_-expressing methanogens in rumen content samples from sheep treated with a methanogen inhibitor. (**A**) Native methanogens counted using spectral flow cytometry in fresh rumen content samples from individual sheep (*n* = 10) were assessed and plotted with methane emission data collected at the same time from the same animal. (**B**) Methanogen numbers were quantified by qPCR using primers to amplify 16S rRNA gene. Data are presented as means of (± SE) of three technical replicates. Animal ID 5009 served as a control and was not treated with inhibitors.

Rumen content samples were collected from control sheep (*n* = 3) and from sheep (*n* = 7) treated with an inhibitor that resulted in complete inhibition of methane production. Low methanogen counts (1.36 × 10^6^) were returned from rumen content samples collected from animals with complete methane inhibition in contrast to high counts (6.20 × 10^8^) in samples from the control animals (Fig. 9A). Methanogen counts obtained using spectral flow cytometry were further confirmed by performing qPCR on the same samples (Fig. 9B).

### Validation of native autofluorescent methanogen counts by mixing rumen content samples

**Fig 10:**
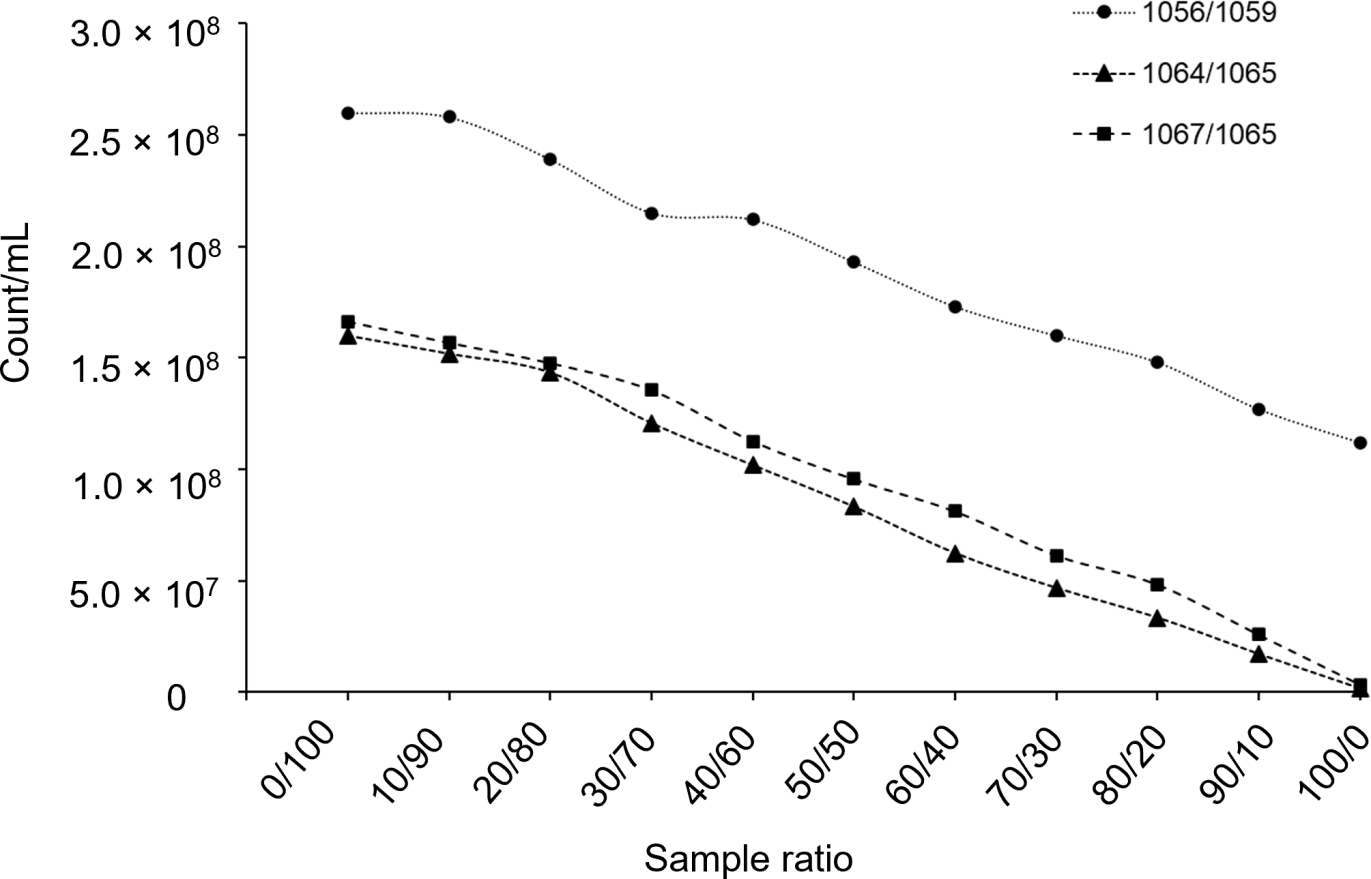
Native F_420_-expressing methanogens in rumen content from mixed cattle rumen content samples. Samples were prepared by mixing different ratios (x-axis) of rumen content samples of the animals showing complete methane inhibition (animal IDs; 1064 and 1067), with intermediate methane inhibition (animal ID; 1056) and untreated-control animals (animal ID; 1065 and 1059). The ratios (e.g., 10/90) are in the same order as the pairs of animal IDs that the samples originated from (shown in the key).

To validate the quantitation method further, cattle rumen content samples from different animals were mixed. Different amounts of rumen content from animals showing complete methane inhibition (animals 1064 or 1067) were mixed with rumen content from cattle showing intermediate methane inhibition (animal 1065). A separate set of samples was prepared by mixing different amounts of rumen content from an animal with partial methane inhibition (animal 1056) with a rumen content sample from a control animal (1059). Samples were then analyzed by spectral flow cytometry, and methanogen counts were plotted against the proportion of the mixed samples (Fig. 10). Our results showed that the methanogen count obtained by spectral flow cytometry varied linearly, as expected, with the proportion of rumen content from the different donor animals.

## DISCUSSION

In this study, we tested a rapid and low-cost flow cytometric method to detect and quantify autofluorescent rumen methanogens. The method requires very little sample preparation and is based on the autofluorescence of the methanogen cofactor F_420_, which is an integral part of their methanogenic pathway. The presence of genes encoding F_420_ biosynthesis enzymes has also been reported in other bacterial and archaeal species (51). However, the concentration of F_420_ in non-methanogenic species has been reported to be approximately 140th of that found in hydrogenotrophic methanogens (52), which would make detection using the current protocol challenging and unlikely to interfere with methanogen counts. The autofluorescence of F_420_ cofactor has been successfully used in flow cytometry to obtain counts of methanogens in the human gut (53) and methanogenic enrichment cultures and digester samples (25). In our study, F_420_ autofluorescence was used to distinguish resident autofluorescent rumen methanogens from other members of the rumen microbial community and from feed particles present in rumen content samples of cattle and sheep. Instead of using conventional flow cytometers, which are based on the principle of employing a dedicated sensor for each fluorescent signal, we used a spectral flow cytometer, which used a wide range of detectors to collect all the fluorescence signals across almost the entire emission spectrum. Spectral flow cytometry offers distinct advantages by using multiple sensors to capture the complete fluorescence signals of a given sample (54). The spectral flow cytometer Cytek Aurora used in this study has three lasers (405 nm, 488 nm, and 640 nm) and 38 fluorescence channels including violet (V1–V16), blue (B1–B14), and red (R1–R8). These features allowed us to measure a wide spectrum of fluorescence from each particle. The increased sensitivity of this spectral flow cytometry method improved the resolution of the rumen methanogen population in comparison to conventional flow cytometry or raw data.

Having identified unique fluorescence profiles for the key particles detected in rumen content, we used a spectral unmixing approach to create unified fluorescence tags: a methanogen tag for rumen methanogen populations and a rumen tag for the rest of the fluorescent rumen content particles. We have found that the unmixing approach gave us a noticeable improvement in signal-to-noise compared to using raw data. We defined the optimal population from rumen content to use as a reference control for unmixing to be “All V12 bright,” as this gave a high staining index score, as well as ease of definition for reproducibility between experiments. This choice of population for reference control was also supported by the %CV of the rumen tag into the methanogen tag from this approach, which remained at comparably low levels, thus minimizing the spread of rumen content signal into the methanogen tag. Our approach further removed the initial ambiguity caused by the presence of particles with higher SSC in the V4 detector from rumen content samples.

The authenticity of the methanogen population that was defined based on intrinsic F_420_ autofluorescence was confirmed by treating individual isolates of cultured methanogens with sodium borohydride (NaBH_4_), which quenches or reduces the cofactor F_420_ (55). The data were compared with non-fluorescent rumen bacterial isolates *R. albus* 7, *S. ruminantium* HD4, *A. hokkaidonensis* R-7, *O. umbonata* Hun279, *Prevotella bryantii* C21a, and *B. proteoclasticus* B316, and non-fluorescent methanogen isolate ISO4-G1. Rumen methanogens were quantified in this study over a broad concentration range of 10^3^–10^8^ methanogens/mL in rumen content samples of cattle and sheep and validated with microscopic counts and qPCR.

Because this method is based on detecting fluorescence from F_420_, it will not detect members of the order *Methanomassiliicoccales*, which make up about 15% of rumen methanogens globally, although their abundance varies depending on the animal’s diet (14). Excluding this group introduces potential bias in abundance estimates and may lead to the underestimation of total methanogen populations when applied in methane mitigation studies. However, targeting *Methanobacteriales* is of interest in the context of vaccine design for methane mitigation, so a tool that focuses on these is of benefit.

The application of this current method for methanogen quantification to rumen content samples collected from animals treated with a methanogen inhibitor demonstrated a real use case of the method. It also provided a way of validating our method using rumen samples that were essentially free of methanogens, as demonstrated by the low counts of archaeal 16S rRNA genes. The signal due to F_420_-containing methanogens was almost absent in rumen samples from cattle and sheep being treated with a methane inhibitor that almost completely abolished methane formation. Mixing rumen contents from animals with differing degrees of methane inhibition resulted in a linear response of methanogen counts proportional to the relative amounts of rumen contents used, as expected from the degrees of methane inhibition from the donor animals. This supports the use of this method to assess the effects of methane mitigation strategies on methanogen counts in the rumen.

Sample preparation is a critical factor in ensuring representative detection of methanogens from rumen content. Many methanogens are closely associated with protozoa and plant particles, which may be underrepresented when analyzing only filtered rumen fluid. Our method employs a 100 µm filter that allows passage of most microbial cells but excludes large particulate matter. Consequently, rumen protozoa typically ranging from 40 µm to over 100 µm and fibrous plant material are likely retained by the filter, preventing clogging of the flow cytometer but introducing a known limitation. To mitigate bias, we collected well-digested rumen contents from housed animals in the morning, rather than immediately post-feeding, to obtain a more homogeneous microbial suspension. This approach reduces large feed particles and better reflects the microbial population in the liquid phase, including free-living methanogens and those loosely associated with smaller particles. Nevertheless, methanogens tightly bound to protozoa or to large feed particles may be underrepresented in flow cytometry-based quantification.

Since methanogenic F_420_ autofluorescence is an integral part of the developed protocol, it was important to verify the stability of F_420_ autofluorescence during the measurement using our experimental conditions and instrument settings. F_420_ autofluorescence can be affected by shifts between the fluorescent oxidized F_420_ and nonfluorescent reduced F_420_H states of the cofactor, as well as by the membrane integrity of fluorescent cells. In addition, fluorescence of cofactor F_420_ can be quenched (55, 56). Aerobic environments have been reported to increase the autofluorescence in methanogens because the nonfluorescent F_420_H, still abundant in the cells, is oxidized. Another important consideration is that some methanogens are vulnerable to oxygen and may be damaged by its influence (18). While we treated and measured our rumen content samples under aerobic conditions, we found that the autofluorescent methanogens (F_420_) were predominantly stable even after 3 days of storage in buffer at 4°C. The relative stability of the autofluorescence signal in rumen content showed that there is no need to analyze samples immediately, and samples can be stored at 4°C for several hours or even for 1 day prior to quantifying methanogens.

Photobleaching can diminish the intensity of the cofactor F_420_ autofluorescence (57), which potentially limits microscopic counting of methanogen cells. In comparison, analysis of cells by flow cytometry only requires exposure of cells to very short periods, that is, 0.75–1.5 μs, and therefore, photobleaching would be minimal and unlikely to bias quantification of methanogenic archaea. Flow cytometry has other advantages as alternative methods, such as DNA sequencing methods, qPCR, fluorescence microscopy, and single-cell labeling by FISH, used for methanogen quantification are often labor intensive and, in some cases, require elaborate data processing (21). In comparison, this spectral flow cytometry assay for methanogen quantification is relatively simple to perform, does not require the addition of chemicals for sample processing and analysis, and results can be obtained relatively quickly. Our data indicate that it can be used as a tool for developing and evaluating potential methane mitigation strategies on ruminant livestock methane emissions.

### Conclusions

Our study showed that autofluorescent rumen methanogenic archaea can be distinguished and quantified by spectral flow cytometry in the complex rumen microbial environment. This is due to the distinctive autofluorescence of rumen content material and its spectral resemblance to a plant sample and the unique spectral profile of the methanogens due to cofactor F_420_. Spectral flow cytometry was able to distinguish between different rumen content particles based on their unique spectral signatures in the rumen, enabling reliable detection and quantification of methanogens in rumen content samples from cattle and sheep over a broad concentration range. We validated this method using samples from animals treated with a methanogen inhibitor that reduced rumen methanogen populations and methane formation to varying degrees.

## Data Availability

Flow cytometry FCS files are available following reasonable request to S.K.

## SUPPLEMENTAL MATERIAL

Supplemental material aem.01416-25-s0001.docx Tables S1 to S4, Text S1, and Fig. S1 to S6.

ASM does not own the copyrights to Supplemental Material that may be linked to, or accessed through, an article. The authors have granted ASM a non-exclusive, world-wide license to publish the Supplemental Material files. Please contact the corresponding author directly for reuse.
