# Supplementary material for: Detection and quantification of rumen methanogens using F420 autofluorescence profiling with spectral flow cytometry
Source: Appl Environ Microbiol. 2026 Feb 9;92(3):e01416-25. doi: 10.1128/aem.01416-25 (PMC12997794; doi:10.1128/aem.01416-25)
Supplement: Supplemental material — Tables S1 to S4, Text S1, and Fig. S1 to S6. [file aem.01416-25-s0001.docx]

**Supplementary Information**

**Detection and quantification of rumen methanogens using F_420_ autofluorescence profiling with spectral flow cytometry**

Sofia Khanum,^1,2,#,^^ Joanna M. Roberts,^3,^^ Maria M. Della Rosa,^2^ Rechelle Sage,^3^ Peter M. Reid,^2^ Priya Soni,^2^ Stefanie Bagley,^1,2^ Stefan Muetzel,^2^ Peter H. Janssen,^1,2^ D. Neil Wedlock^1,2^

^1^Lucidome Bio Ltd, New Zealand

^2^AgResearch Group, Bioeconomy Science Institute, Palmerston North, New Zealand

^3^Flowjoanna, Palmerston North, New Zealand

^#^Correspondence to: Sofia Khanum; [sofia.khanum@agresearch.co.nz](mailto:sofia.khanum@agresearch.co.nz)

^^^Sofia Khanum and Joanna M. Roberts contributed equally to this work.

**Table S1.** Details of animals measured and sampled in the cattle trial.

| Animal ID | Diet | CH_4_ inhibition* |
| --- | --- | --- |
| 1048 | Fresh ryegrass | Full |
| 1049 | Fresh ryegrass | Full |
| 1050 | Ryegrass based baleage | Full |
| 1052 | Fresh ryegrass | Full |
| 1053 | Ryegrass based baleage | Full |
| 1054 | Ryegrass based baleage | Full |
| 1064 | Fresh ryegrass | Full |
| 1066 | Fresh ryegrass | Full |
| 1067 | Ryegrass based baleage | Full |
| 1069 | Ryegrass based baleage | Full |
| 1070 | Ryegrass based baleage | Full |
| 1071 | Fresh ryegrass | Full |
| 1055 | Fresh ryegrass | Partial |
| 1065 | Ryegrass based baleage | Partial§ |
| 1068 | Fresh ryegrass | Partial |
| 1056 | Ryegrass based baleage | No |
| 1058 | Fresh ryegrass | No |
| 1059 | Fresh ryegrass | No |
| 1060 | Ryegrass based baleage | No |
| 1062 | Fresh ryegrass | No |
| 1063 | Ryegrass based baleage | No |
| 1057 | Ryegrass based baleage | No (true control) |
| 1061 | Fresh ryegrass | No (true control) |

*Cattle were classified as: a) *No CH_4_ inhibition*: CH_4_ yield similar to animals receiving no inhibitor (*true controls*), negligible hydrogen release, no formate or primary/secondary alcohols detected in rumen fluid, no increase of propionate and valerate proportion in rumen fluid; b) *Partial CH_4_ inhibition*: 20% to 50% of CH_4_ yield decrease compared to animals that received no inhibitor, hydrogen release above 0.4 g/kg dry matter feed intake, formate detected and increased propionate and valerate proportions in rumen fluid; c) *Full CH_4_ inhibition*: CH_4_ yield decrease of 98% to 100% compared to the animals that received no inhibitor, hydrogen release above 2 g/kg dry matter feed intake, formate detected and increased propionate and valerate proportions in rumen fluid.

^§^Considered as partial CH_4_ inhibition due to the changes observed in rumen fermentation even though no hydrogen was released.

**Table S2.** Details of animals measured and sampled in sheep trial.

| Animal ID | Diet | CH_4_ inhibition* |
| --- | --- | --- |
| 5001 | Fresh ryegrass | Full |
| 5002 | Fresh ryegrass | No |
| 5003 | Fresh ryegrass | No |
| 5004 | Fresh ryegrass | Full |
| 5005 | Fresh ryegrass | Full |
| 5006 | Fresh ryegrass | Full |
| 5007 | Fresh ryegrass | Full |
| 5008 | Fresh ryegrass | Full |
| 5009 | Fresh ryegrass | No |
| 5010 | Fresh ryegrass | Full |

*The criteria for inhibition were the same as those used in Supplementary Table S1.

**Table S3.** Configurations for use of Cytek® Aurora cytometer. The Cytek® Aurora 3L configuration was equipped with a 100-mW violet 405 nm laser, a 50-mW blue 488nm laser and 80-mW red 640nm laser. The optical configuration included three scatter detectors (FSC and SSC from the 488nm laser and SSC from the 405nm laser). The detector array for each laser was comprised of several avalanche photodiodes with a range of fluorescence filters to capture close to the full emission spectrum of fluorescent light from each laser. The detector channel number and associated bandpass fluorescent filter are shown in this table.


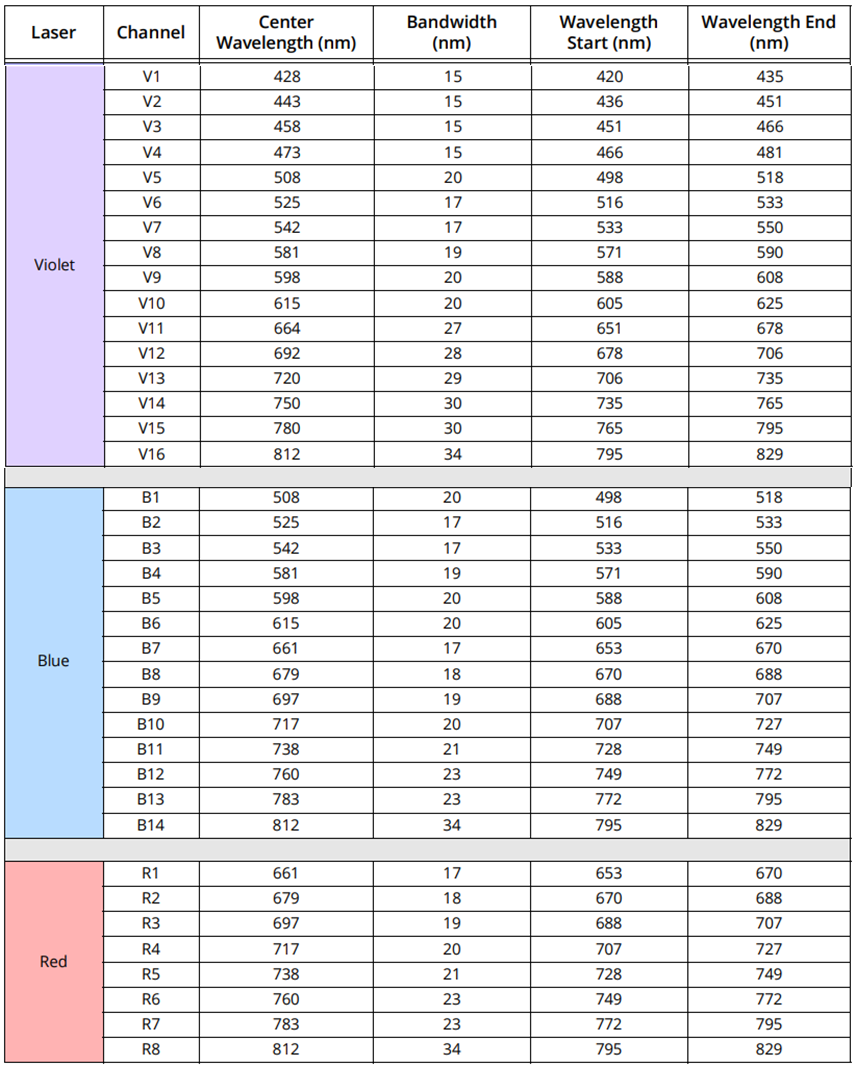


Adapted from Cytek® Aurora website (<https://www.embl.org/groups/flow-cytometry-heidelberg/wp-content/uploads/2021/10/Cytek-Aurora-Configuration.pdf>).

**Table S4.** Statistical error of the mean for flow cytometry counts across serial dilutions.

| Mean no. of cells/mL determined by microscopy | Mean no. of cells/mL determined by flow cytometry | Statistical error fraction | Statistical error (%) |  |
| --- | --- | --- | --- | --- |
| 4.29 × 10^1^ | 17 | 4.123 | 24.3 |  |
| 4.29 × 10^2^ | 15 | 3.916 | 25.5 |  |
| 4.29 × 10^3^ | 77 | 8.794 | 11.4 |  |
| 4.29 × 10^4^ | 613 | 24.752 | 4.0 |  |
| 4.29 × 10^5^ | 5989 | 77.391 | 1.3 |  |
| 4.29 × 10^6^ | 57108 | 238.973 | 0.4 |  |
| 4.29 × 10^7^ | 515000 | 717.635 | 0.1 |  |

**Text S1. Gain adjustments for the Cytek® Aurora cytometer used to quantify methanogens in rumen content**

A SSC threshold (violet laser) was adjusted so that the events per second (eps) from a sterile PBS sample were below 500 (typically around 180 eps) and were primarily from noise associated with the sheath fluid and electronics, rather than the PBS sample (determined by adjusting the flow rate between low, medium and high and seeing minimal difference in eps). The Cytek Assay settings on Aurora were adjusted across all three lasers with a 200% gain increase. Samples were acquired using low flow rate and the aborts per second were monitored with concentrated samples to ensure they were below 5-10% of eps. If a sample was above this level, it was diluted further or not used.

**
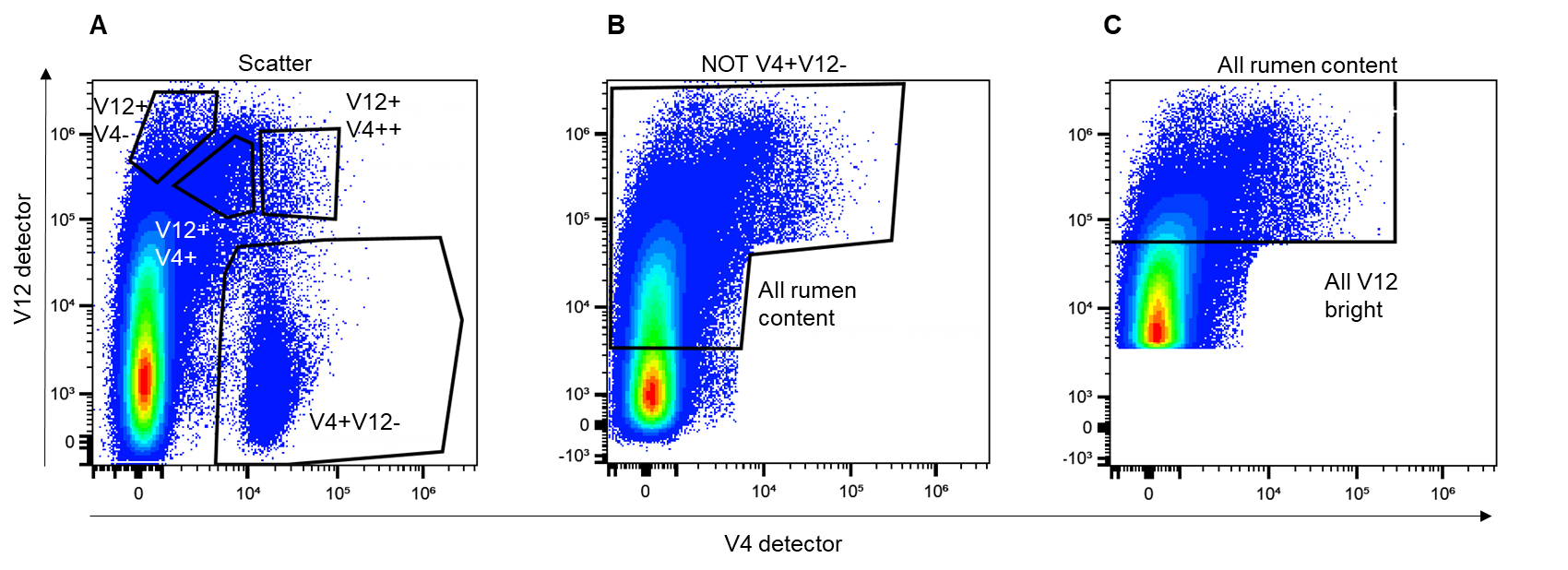
**

**Fig S1.** To determine the optimal strategy for unmixing rumen contents in the Spectroflo^TM^ unmixing algorithm, various regions in rumen contents were defined based on levels of signal in the V12 and V4 detectors, to test individually as the “reference” material for the rumen content tag in spectral unmixing. In (**A)**, three regions, each capturing a unique segment of rumen contents, are shown: V12+V4-, V12+V4+, V12+V4++; each were tested in unmixing workflows (the V4+ V12- region is enriched for rumen methanogens). In **(B)** and **(C)**, plots are shown to demonstrate how a fourth rumen content region is delineated for testing in unmixing, first defined based on a boolean “Not” gate on V4+ V12-, and then on all rumen content. A fifth rumen content region is defined as a subset of that gate, called All V12 bright.


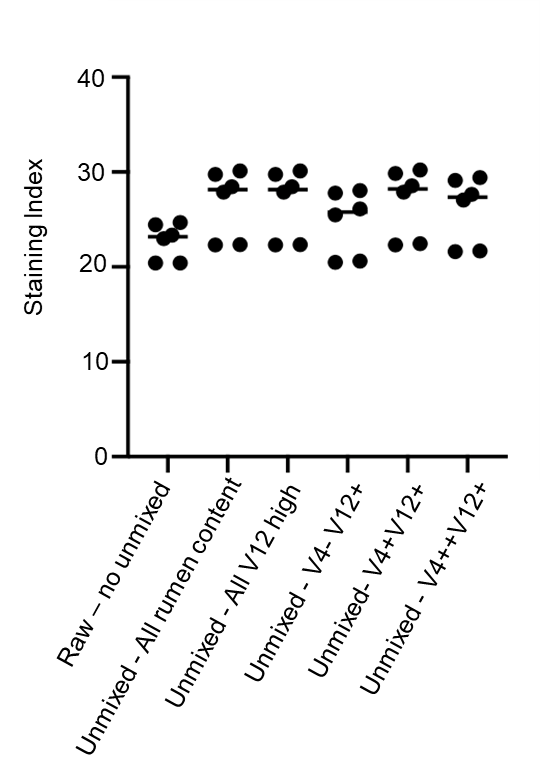


**Fig S2.** Comparison of signal strength in rumen contents using staining index calculations to assess the effectiveness of different reference controls (as defined in Supplementary Figure 1) for optimal spectral unmixing. The lowest staining index from raw data (not unmixed) was seen, while the least effective rumen content reference control to use with unmixing was V4− V12+. Others are reasonably similar. Duplicates from three separate rumen content samples are shown.

**
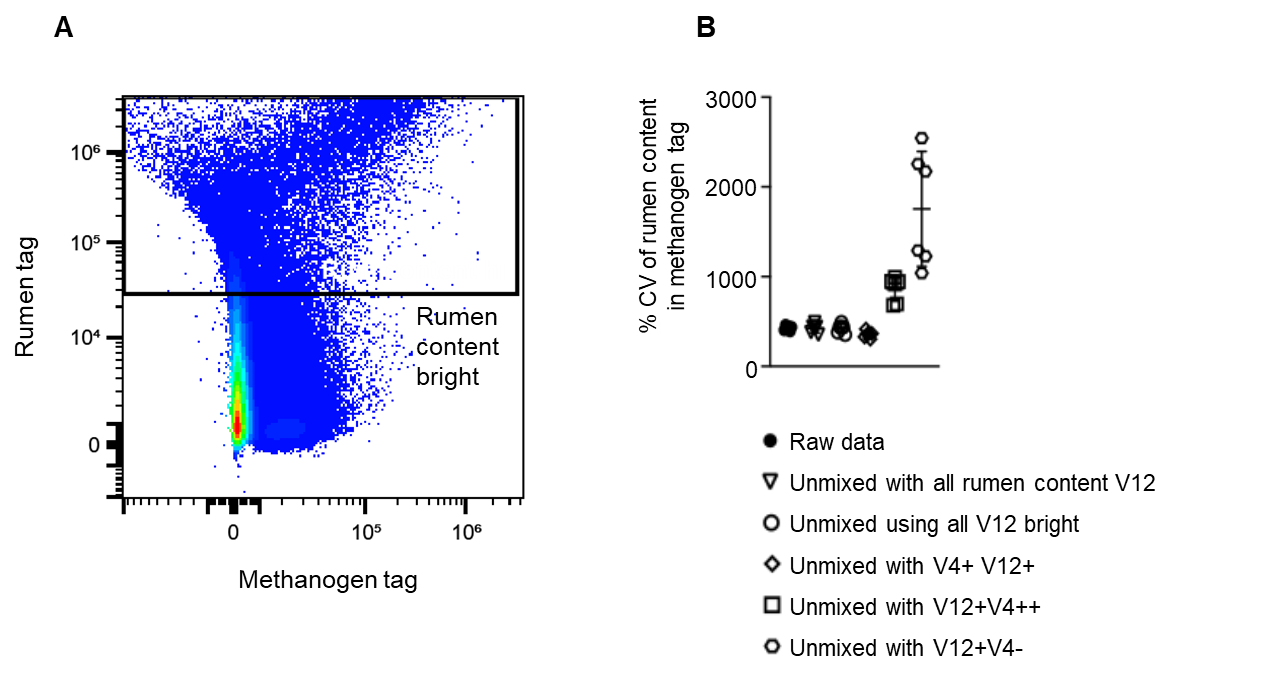
**

**Fig S3.** Methanogen tag %CV of rumen content particles to compare unmixing approaches. (A) rumen content particles used for unmixing (B) %CV determined for rumen content particles (rumen content high), for all the unmixing strategies with different cells for rumen content reference control, as indicated on x axis on graph. Raw and unmixed “all rumen content V12”, “All V12 high”, “V4+V12+” and “V12+V4++” gave lower variances, while unmixed “V12+ V4-” gave highest variance.


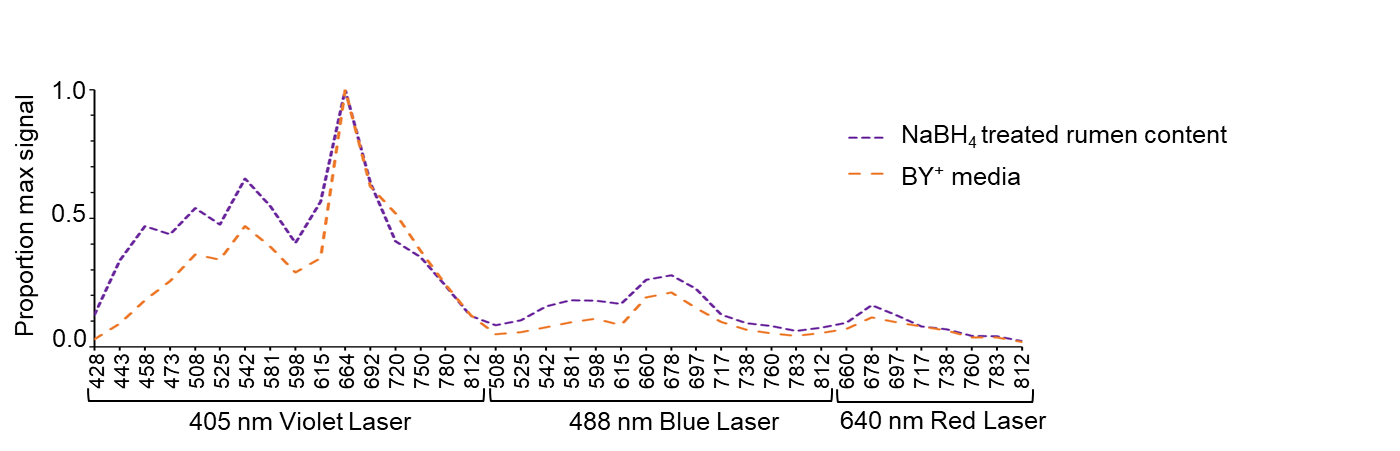


**Fig S4.** A BY^+^ medium-only sample and a rumen content sample treated with NaBH_4_ were analysed by spectral flow cytometry. The median fluorescence signal intensity for each fluorescent filter/detector was measured and expressed as a proportion of the maximal signal. Normalizing the median fluorescent signals revealed that a BY^+^ medium sample, which contains 5% clarified rumen content, and a rumen content sample treated with NaBH_4_ to quench autofluorescence of rumen methanogens have an almost identical spectral signature. This analysis confirmed that the particles in the BY^+^ medium are likely the source of the additional “non methanogen” particles counted in cultured methanogen samples.


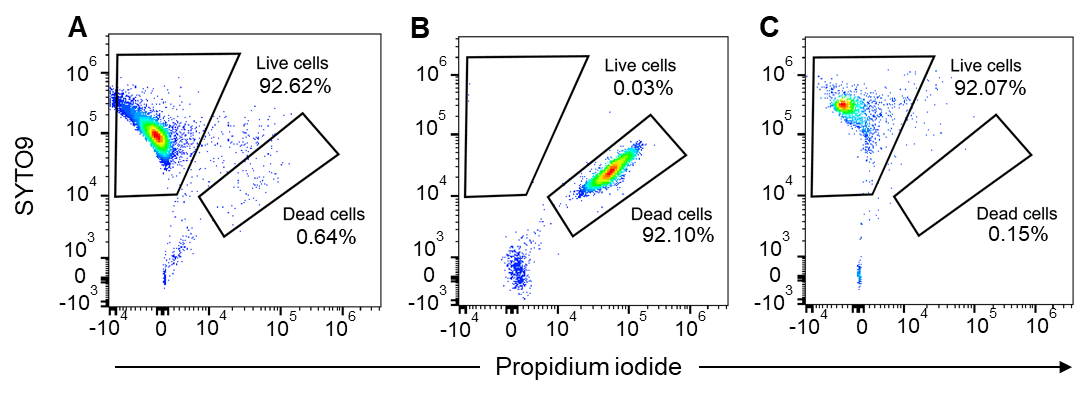


**Fig S5.** Cell membrane integrity of NaBH_4_-treated cultured methanogens. *Methanobrevibacter ruminantium* M1 cells were treated with SYTO9 (membrane permeable) and propidium iodide (propidium iodide; membrane impermeable) dyes and then measured using spectral flow cytometry. The 508 nm (B1) detector was used as the primary channel for the live (SYTO9 positive) cells and the 660 nm detector (B7) used as the primary channel for the dead (propidium iodide negative) populations of M1 cells. A live cells gate was created for the live cell population based on their SYTO9 fluorescence intensities on the y-axis. A dead cells gate was created based on the propidium iodide fluorescence intensities on the x-axis. **(A)** M1 cells stained with SYTO9 and propidium iodide, **(B)** M1 cells treated with 70% isopropanol and stained with SYTO9 and propidium iodide, **(C)** M1 cells treated with NaBH_4_ and stained with SYTO9 and propidium iodide.

**
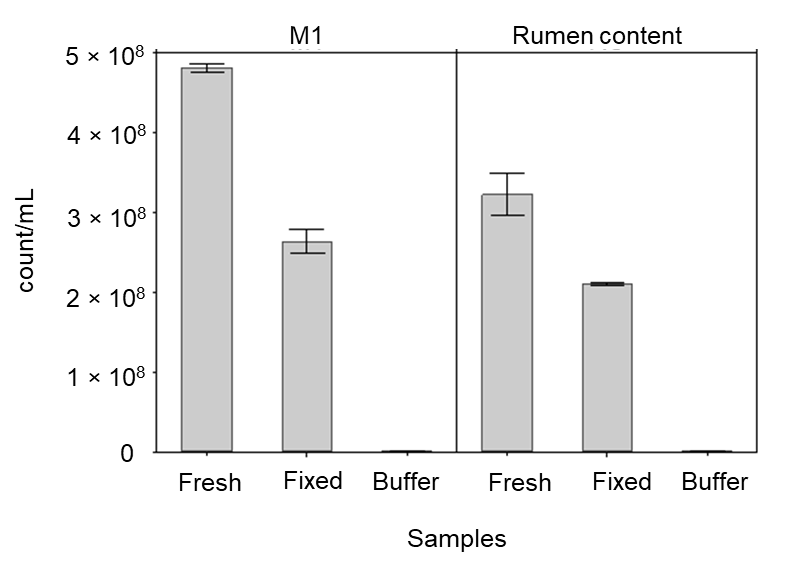
**

**Fig S6.** Stability of methanogen counts after fixation, determined using spectral flow cytometry. Cultured *Methanobrevibacter ruminantium* M1 cells and rumen content samples were fixed with 2% (v/v) paraformaldehyde for 30 min. Autofluorescent methanogens in fresh and fixed samples and in buffer were quantified by spectral flow cytometry. Error bars represent the standard deviation of three technical replicates. Buffer is the PBS suspension buffer used without any added methanogens.
